# CiliOPD: a ciliopathy-associated COPD endotype

**DOI:** 10.1186/s12931-021-01665-4

**Published:** 2021-02-27

**Authors:** Jeanne-Marie Perotin, Myriam Polette, Gaëtan Deslée, Valérian Dormoy

**Affiliations:** 1 University of Reims Champagne-Ardenne, Inserm, P3Cell UMR-S1250, SFR CAP-SANTE, CHU Maison Blanche, 45 rue Cognacq-Jay, 51092 Reims, France; 2 Department of Respiratory Diseases, CHU of Reims, Hôpital Maison Blanche, 51092 Reims, France; 3 Department of Biopathology, CHU Reims, Hôpital Maison Blanche, 51092 Reims, France

**Keywords:** COPD, Cilia, Transcriptomic

## Abstract

The pathophysiology of chronic obstructive pulmonary disease (COPD) relies on airway remodelling and inflammation. Alterations of mucociliary clearance are a major hallmark of COPD caused by structural and functional cilia abnormalities. Using transcriptomic databases of whole lung tissues and isolated small airway epithelial cells (SAEC), we comparatively analysed cilia-associated and ciliopathy-associated gene signatures from a set of 495 genes in 7 datasets including 538 non-COPD and 508 COPD patients. This bio-informatics approach unveils yet undescribed cilia and ciliopathy genes associated with COPD including NEK6 and PROM2 that may contribute to the pathology, and suggests a COPD endotype exhibiting ciliopathy features (CiliOPD).

## Introduction

Cilia dysfunction is a hallmark of chronic obstructive inflammatory lung diseases [1]. Alterations of both cilia structure and function alter airway mucociliary clearance. Epithelial remodelling is indicted in COPD pathogenesis, including distal to proximal repatterning of the small airways and altered generation of motile and primary ciliated cells [2–4].

Cellular processes related to cilia dysfunction such as autophagy [5] may represent therapeutic targets, although the genetic print of cilia involvement in COPD has only been observed in comparative gene expression studies providing COPD- and smoking-associated signatures [6–8]. In this study, we thought to investigate further cilia dysregulation in COPD. Rather than focussing on the biological samples to identify the most significant hits across the whole genetic code, we comparatively analysed cilia-associated and ciliopathy-associated gene signatures in 7 datasets including 538 non-COPD and 508 COPD patients.

## Methods

### Gene selection

Human cilia-associated genes (n = 447) and ciliopathy-associated genes (n = 189) were extracted from the reviewed (Swiss-Prot) records [9] combined with the data of the 100,000 Genomes Project [10], and the four compiled public libraries “CentrosomeDB” [11], “CilDB” [12], “SysCilia” [13], and “CiliaCarta” [14] as previously described [15] for a total of 495 unique entries defined as cilia geneset (Additional file 1: Table S1). In brief, 2 queries were entered on UniProtKB to extract reviewed entries in Human (https://www.uniprot.org; date of retrieval July 27th 2020): (i) cilia-associated genes: “cilia AND reviewed:yes AND organism:"Homo sapiens (Human) [9606]"” yielded 447 entries; (ii) ciliopathy-associated genes: “ciliopathy AND reviewed:yes AND organism:"Homo sapiens (Human) [9606]"” yielded 171 entries. Both lists of genes were compared and completed with the lists of cilia-associated genes and ciliopathy-associated genes described in the data from the 100,000 Genomes Project, CentrosomeDB, CilDB, SysCilia, and CiliaCarta.

### RNAseq data analysis

Previously published datasets of gene expression of whole lung tissue samples and small airways bronchoscopic samples obtained from non-COPD and COPD patients, that were available online, were collected (GEO database; http://www.ncbi.nlm.nih.gov/geo; accession numbers: GSE47460, GSE57148, GSE76925, GSE103174, GSE11784, GSE37147, GSE56341). The expression of the genes of interest (human cilia-associated genes and ciliopathy-associated genes) was analysed depending on COPD status and expressed as fold-change compared to non-COPD. Associations with clinical/functional characteristics were searched. Analyses were performed separately in the lung compartment (“whole lung tissue sample”, GSE47460, GSE57148, GSE76925, GSE103174) and in small airway epithelial cells (SAEC) (“small airways bronchoscopic samples”, GSE11784, GSE37147, GSE56341).

GSE plots contain all the genes of the genesets for each dataset. The y-axis is the log10 ratio obtained by dividing the mean of each gene’s value in lung tissues or SAEC of non-COPD patients by its value in COPD patients. The Venn Diagram were designed with a tool from VIB/UGent (http://bioinformatics.psb.ugent.be/webtools/Venn).

### Single cell sequencing analysis

The published dataset can be found on http://www.lungcellatlas.org. We retained cell clustering based on the original studies and considered only subjects with no respiratory disease [16].

### Statistics

Comparisons of transcriptomic data between COPD and non-COPD subjects were performed within each dataset using SPSS v24. Paired t-tests were applied to the log2 transformed transcriptomic data; p < 0.05 was considered significant. False discovery rate (FDR) correction was applied.

## Results

**Fig. 1:**
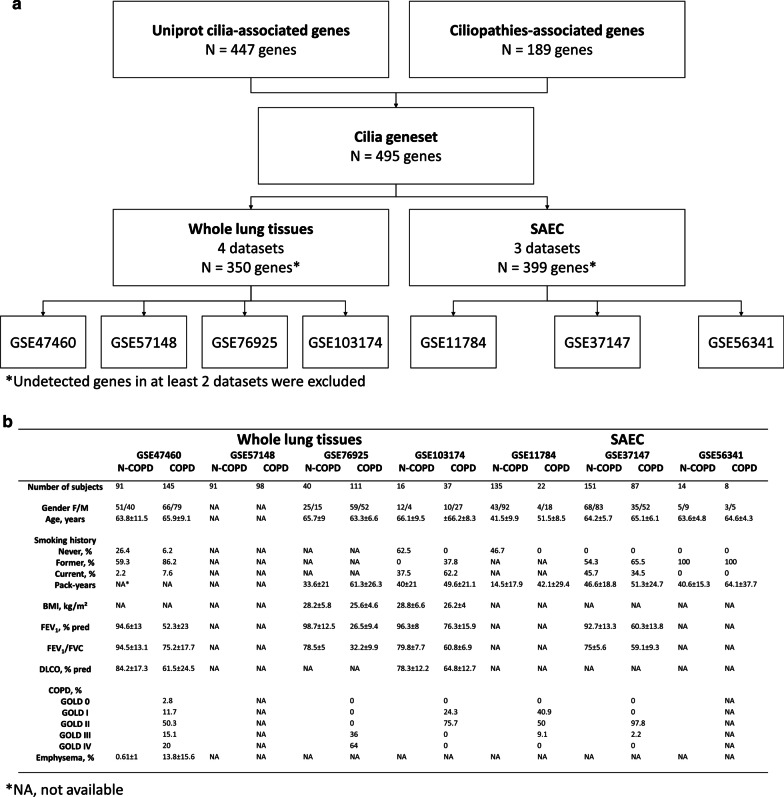
Study design. **a** Flow chart defining the selection of cilia/ciliopathy-associated genes analysed in the 4 whole lung tissues and 3 SAEC datasets. **b** Table summarizing the available clinical parameters across the 7 datasets in non-COPD (N-COPD) and COPD patients

A total of 7 public datasets of transcriptomic analysis of either whole-lung tissues (238 non-COPD and 391 COPD patients) or SAEC (300 non-COPD and 117 COPD patients) were analysed to identify an alteration of cilia- and ciliopathy-associated genes expression in COPD patients when compared with non-COPD patients (Fig. 1a, b, Additional file 1: Table S1).

**Fig. 2:**
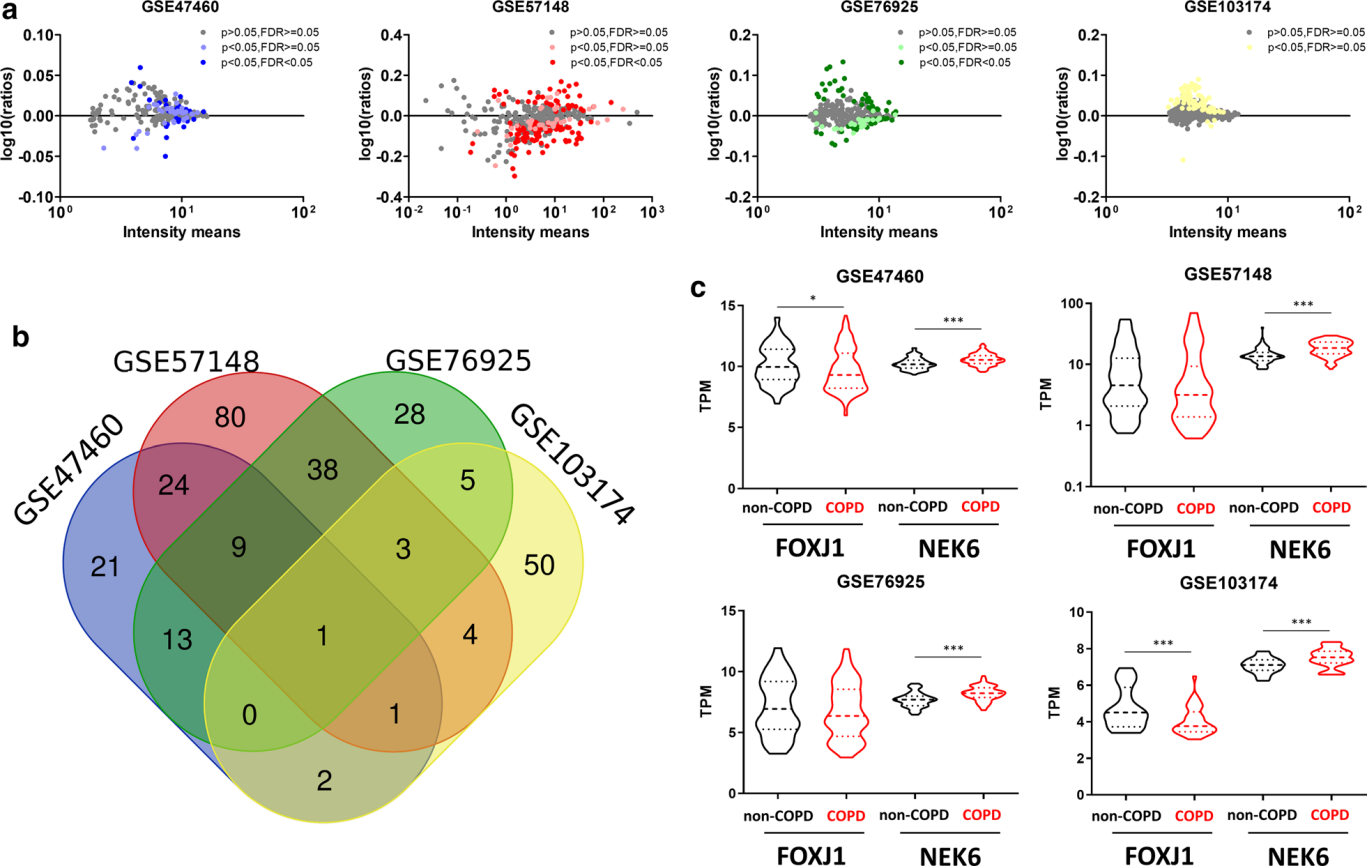
Identification of cilia-associated deregulated genes in COPD whole lung tissues. **a** dot plots showing cilia-associated genes signature quantification and significant fold change in COPD lung tissues compared with non-COPD lung tissues per dataset. **b** Venn diagram of overlapping genes between the 4 datasets of whole-lung tissues regarding the 350 cilia-associated genes. **c** Truncated violin-plots showing mean and IQR for the expression levels of FOXJ1 and NEK6 for the 4 whole lung tissue datasets. *, p < 0.05; ***, p < 0.001 non-COPD vs COPD

Considering the 4 datasets obtained from whole-lung tissues, 71/160/97/66 genes were significantly deregulated in COPD patients representing respectively 26%/42%/30%/17% of the 350 genes in the whole lung cilia geneset (Fig. 2a and Additional file 1: Tables S2–S5). Fourteen deregulated genes were identified in at least 3 datasets, 100 in at least 2 datasets, and 279 in at least 1 dataset, corresponding to respectively 4%/29%/80% of genes in the whole lung cilia geneset (Fig. 2b and Table 1). We then focused on the 14 commonly dysregulated genes, and confirmed the localization of the proteins and the expression of the transcripts in AEC (Additional file 1: Figure S1 and Table 1). Among those 14 genes, 4 have been identified as associated with COPD in previous studies (BBS9, GLI2, NEK6, WDPCP) [6, 17–20]. NEK6 was significantly upregulated in the 4 datasets in COPD patients (Fig. 2c and Table 1).

**Table 1:** List of the main commonly deregulated cilia/ciliopathy-associated genes (n = 24) in COPD patients

Genes Ids	Uniprot Id	Gene name	Gene expression (Log2 fold change vs non-COPD)				Ref	HPA^a^	SC^b^	
Whole lung			GSE47460	GSE57148	GSE76925	GSE103174			%	P
BBS7	Q8IWZ6	Bardet-Biedl syndrome 7 protein		0.21	− 0.29	− 0.06		MCC	+	All
BBS9	Q3SYG4	Bardet-Biedl syndrome 9 protein	− 0.01	0.13	0.04		[6]	MCC	+ +	MCC
CALM3	P0DP25	Calmodulin-3	0.02	0.11	0.03			MCC	+ + +	All
CEP78	Q5JTW2	Centrosomal protein of 78 kDa		0.10	0.10	− 0.05		NDC	+ +	MCC
DZIP1	Q86YF9	DAZ-interacting protein ½	0.02	0.22	0.11			MCC	+ +	MCC
GLI2	P10070	GLI family zinc finger protein 2	0.04	0.20	0.20		[17, 18]	All	+	All
NEK6	Q9HC98	Never in mitosis A-related kinase 6	0.05	0.40	0.09	0.08	[19]	EC	+	All
NUBP2	Q9Y5Y2	Nucleotide-binding protein 2	− 0.01	− 0.56	0.09			All	+ +	All
PDE6D	O43924	Retinal rod rhodopsin-sensitive cGMP 3′,5′-cyclic phosphodiesterase subunit delta	0.01	− 0.17	0.03			All	+	All
PRPF4	O43172	U4/U6 small nuclear ribonucleoprotein Prp4		0.15	0.03	0.04		All	+	All
PRPF8	Q6P2Q9	Pre-mRNA-processing-splicing factor 8	− 0.01	0.34	0.02			All	+ +	All
PTPDC1	A2A3K4	Protein tyrosine phosphatase domain-containing protein 1	− 0.02	0.12	0.04			NDC	+	All
TMEM138	Q9NPI0	Transmembrane protein 138	0.02	− 0.18	0.06			MCC	+ +	MCC
WDPCP	095876	WD repeat-containing and planar cell polarity effector protein fritz homolog	− 0.02	0.21		− 0.09	[20]	EC	+ +	MCC

Genes Ids	Uniprot Id	Gene name	Gene expression (Log2 fold change vs non-COPD)				Ref	HPA^a^	SC^b^	
Small airway epithelial cells (SAEC)			GSE11784	GSE37147	GSE56341				%	P
ADAM15	Q13444	Disintegrin and metalloproteinase domain-containing protein 15		0.04	0.07		[21, 24]	MCC	+	All
ADCY6	O43306	Adenylate cyclase type 6		0.02	0.02			MCC	+	EC
ANKMY2	Q8IV38	Ankyrin repeat and MYND domain-containing protein 2	0.35	− 0.01				ND	+	All
ARMC4	Q5T2S8	Armadillo repeat-containing protein 4		− 0.02	− 0.02			MCC	+ + +	MCC
BBS4	Q96RK4	Bardet-Biedl syndrome 4 protein		− 0.03	− 0.03			EC	+ +	MCC
DCDC2	Q9UHG0	Doublecortin domain-containing protein 2		− 0.03	− 0.05		[22, 23]	All	+ +	MCC
EFCAB2	Q5VUJ9	Dynein regulatory complex protein 8		− 0.03	− 0.05			MCC	+ + +	MCC
PROM2	Q8N271	Prominin-2	0.55	0.04	0.06			EC	+ +	EC
TTC23	Q5W5X9	Tetratricopeptide repeat protein 23		− 0.02	− 0.04			All	+	All
WDR34	Q96EX3	Cytoplasmic dynein 2 intermediate chain 2	0.40	0.01			[6]	EC	+ + +	MCC

^a^HPA, Human Protein Atlas protein localization (cell population)

^b^SC, Single cell transcriptomic signature: % (percentage of cell expressing the transcript of interest; + , low (< 25%); +  + , intermediate (25–50%); +  +  + , high (> 50%)) and P (principal cell population expressing the transcript of interest); *MCC* multiciliated cells, *NDC* non-differentiated cells, *EC* epithelial cells, All, epithelial and non-epithelial cells; *ND* not detected

**Fig. 3:**
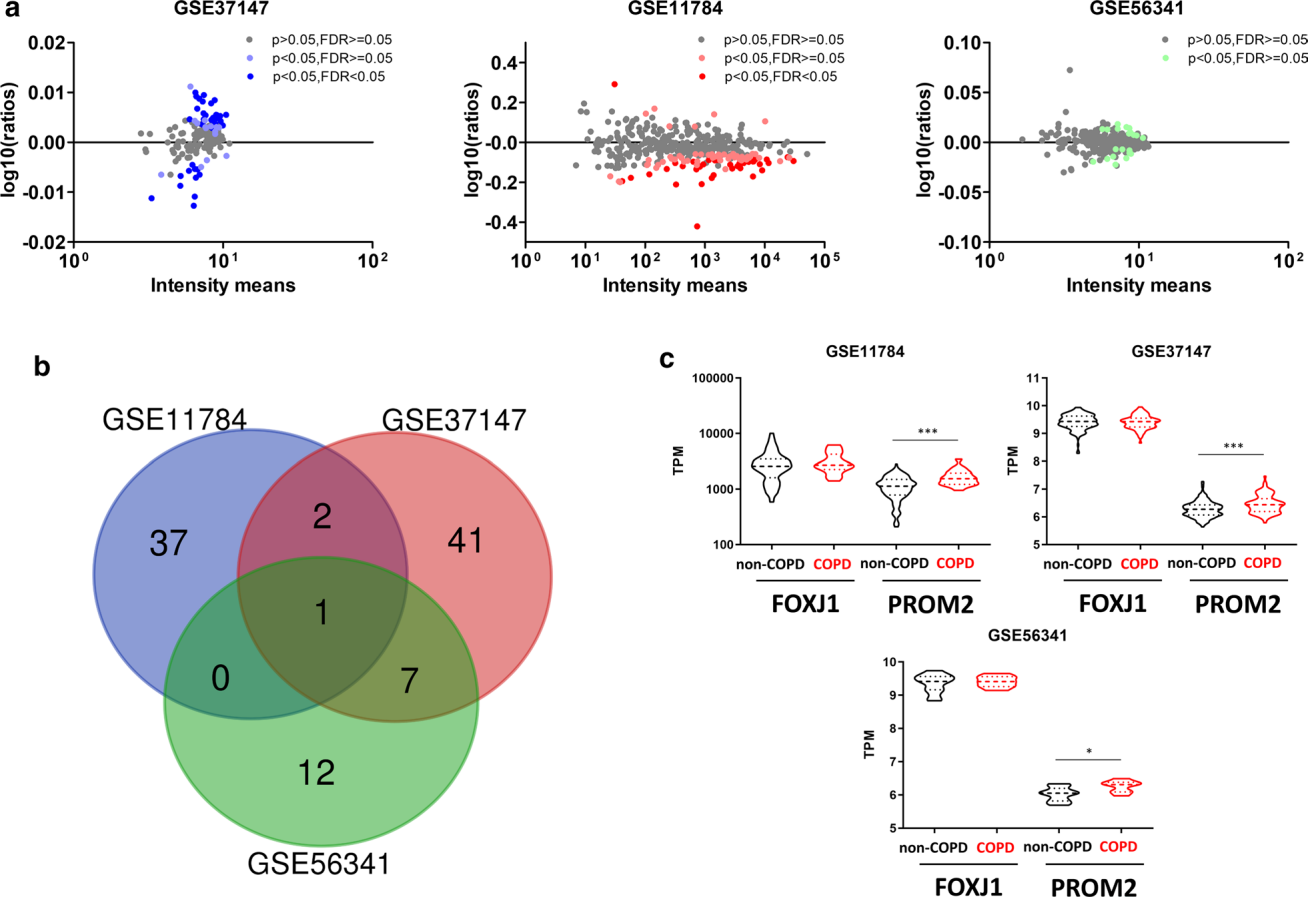
Identification of cilia-associated deregulated genes in COPD SAEC. **a** Dot plots showing cilia-associated genes signature quantification and significant fold change in COPD SAEC compared with non-COPD SAEC per dataset. **b** Venn diagram of overlapping genes between the 3 datasets of SAEC regarding the 399 cilia-associated genes. **c** Truncated violin-plots showing mean and IQR for the expression levels of FOXJ1 and PROM2 for the 3 SAEC datasets. *, p < 0.05; ***, p < 0.001 non-COPD vs COPD

Considering the 3 databases from SAEC, 40/51/22 genes were significantly deregulated in COPD patients representing respectively 10%/33%/5% of the 399 genes in the SAEC cilia geneset (Fig. 3a and Additional file 1: Tables S6–S8). Ten deregulated genes were identified in at least 2 datasets, 100 in at least 1 datasets, corresponding to respectively 2.5%/25% of genes in the SAEC geneset (Fig. 3b and Table 1). Focusing on the 10 commonly dysregulated genes, we confirmed the localization of the proteins and the expression of the transcripts in AEC (Additional file 1: Figure S2 and Table 1). Among those 10 genes, 3 have been identified as associated with COPD in previous studies (ADAM15, DCDC2, WDR34) [6, 21–24]. PROM2 was significantly upregulated in the 3 datasets in COPD patients (Fig. 3c and Table 1).

We identified 23 genes deregulated in COPD in at least 2 whole-lung datasets and 1 SAEC dataset (Additional file 1: Figure S3A and B), 12 were associated to ciliopathies (Additional file 1: Figure S3B and C) representing 6% of ciliopathy-associated genes. In addition, 47% of ciliopathy-associated genes (n = 88) were found deregulated in either COPD lung tissues or SAEC.

## Discussion

Since a few cilia-associated genes were found enriched in GWAS, impaired ciliary function has been suggested to contribute to the pathogenesis of COPD [25]. Here, we considered the whole spectrum of cilia-associated genes and genes involved in known ciliopathies to compare their expression levels between COPD and non-COPD patients. The novelty of our approach lies in the concept that the alteration of cilia is paramount in COPD pathogenesis and that this organelle and its alterations are directly involved in COPD pathophysiology rather than simple collateral damage. We identified a dysregulation of the expression of 29% of cilia genesets in lung tissues and 16% in SAEC in COPD patients, suggesting that an alteration of cilia structure and/or function is an important feature of COPD. Previous comparative genetic studies on COPD patients identified a few genes associated to cilia. In this study, we revealed the full extent of cilia dysregulated expression and we questioned the genetic print of ciliopathies connected to COPD. Further studies will need to assess the role of dozens of candidate that may be functionally involved in the pathophysiology of COPD.

Motile cilia are located in the airways up to the respiratory bronchioles. Primary cilia are observed on non-differentiated epithelial cells, fibroblasts, smooth muscle cells, and endothelial cells [1, 4]. Thus, an alteration of cilia-associated genes may greatly impact the functions of the main pulmonary cell populations. We identified here two sets of dysregulated cilia-related genes depending on the initial tissue sampling: whole lung tissue including all lung cell populations, and SAEC restricted to the epithelial tissue. The 177 genes deregulated in COPD patients in at least 3 datasets (2 whole lung and 1 SAEC) were mainly involved in biological processes associated to ciliary retrograde and anterograde transport, Hedgehog signalling, cilium beat frequency, cilium assembly and microtubule anchoring at centrosome. Since a large quantity of genes orchestrating cilia formation and function were found altered in COPD, we evidence a global cilia dysfunction at the root of the disease rather than a punctual alteration observed as a consequence of pathophysiological mechanisms.

Among the candidate genes, we identified for the first time a list of 24 genes (Table 1) commonly deregulated in the majority of datasets in COPD patients. The impacted biological processes corresponded to the aforementioned ones, suggesting that these genes may represent key actors to understand cilia dysfunction in COPD. Interestingly, 7 genes have been previously highlighted in experimental investigations in the context of COPD [6, 17–24]. Although they were often fortuitously exposed, their associations with COPD were found sufficiently significate to be mentioned. Single-nucleotide polymorphisms (SNP) predicting alteration of lung function were identified for DCDC2 and WDPCP in GWAS [20, 22, 23]. Transcriptomic studies unveiled an upregulation of NEK6 in COPD patients, a downregulation of BBS9 in large airway epithelial cells (LAEC) of smokers compared to non-smokers but not in SAEC, and a downregulation of WDR34 in SAEC of smokers compared to non-smokers but not in LAEC [6, 19]. In vitro and in vivo approaches unveiled differential protein expression and localization for ADAM15 and GLI2 according to cell populations (epithelial cells vs non-epithelial cells or differentiated epithelial cells vs non-differentiated epithelial cells) and sub-cellular localization (cytoplasm vs nuclear) [17, 18, 21, 24]. These findings were generally concordant to the relative gene expression levels we reported across multiple genesets. Further investigations will necessarily require analysis of every components of the molecular print of each candidate in order to recognize its interest in COPD studies including but not limited to: SNP and copy number alterations, gene expression levels, and cellular and sub-cellular protein localizations.

In addition, the expressions of 2 genes were associated with COPD status in all 4 lung databases (*NEK6*, coding for a serine/threonine-protein kinase involved in cell cycle progression during M phase) and in all 3 SAEC databases (*PROM2,* coding for a transmembrane glycoprotein involved in intracellular trafficking), both being upregulated in COPD. Using single cell sequencing analysis, we confirmed that all commonly dysregulated genes (14 in lung datasets and 10 in SAEC datasets) were mainly expressed by multiciliated cells (motile cilia) and non-differentiated epithelial cells (primary cilia) (Table 1).

This hypothesis-generating study has limitations: the lack of clinical data available did not allow us to perform any analysis of the contribution of smoking history or associations with COPD severity; our analysis focused on cilia/ciliopathy-associated genes but hundreds more are involved in centriole regulation and could potentially participate to cilia alterations, nonetheless it would be challenging to distinguish their involvement during interphase (centrosome) and quiescence (ciliogenesis). Although experimental validation of the gene candidates that we identified will be needed, our results suggest alteration of cilia-related cellular and tissular processes that might have a role in COPD pathophysiology.

Ciliopathies refer to genetic disorders that are caused by the abnormal formation or function of cilia. Since cilia and cilia-associated genetic signature are abnormal in COPD patients, COPD could be included in the spectrum of ciliopathies and ciliopathy-associated COPD (CiliOPD) may be considered as a COPD endotype.

## Supplementary Information

**Additional file 1.** Additional figures and tables.

## Data Availability

All data generated or analyzed during the current study are available from the corresponding author on reasonable request.

## Abbreviations

COPD: Chronic obstructive pulmonary disease
SAEC: Small airway epithelial cells
GEO: Gene Expression Omnibus
NEK6: NIMA-related kinase 6
PROM2: Prominin-2
GWAS: Genome wide association study
SNP: Single-nucleotide polymorphism
LAEC: Large airway epithelial cells
