# Supplementary material for: CiliOPD: a ciliopathy-associated COPD endotype
Source: Respir Res. 2021 Feb 27;22:74. doi: 10.1186/s12931-021-01665-4 (PMC7912836; doi:10.1186/s12931-021-01665-4)

**Additional information**

**COPD or CiliOPD**

Jeanne-Marie Perotin, Myriam Polette, Gaëtan Deslée, Valérian Dormoy

**Table of content**

[**1.** **Additional information Tables** 2](#_Toc60660473)

[**Table S1. List of cilia/ciliopathy-associated genes in the cilia geneset (n=495).** 2](#_Toc60660474)

[**Table S2. List of deregulated cilia/ciliopathy-associated genes (n=278) in COPD whole lung tissues: GSE47460** 13](#_Toc60660475)

[**Table S3. List of deregulated cilia/ciliopathy-associated genes (n=378) in COPD whole lung tissues: GSE57148** 15](#_Toc60660476)

[**Table S4. List of deregulated cilia/ciliopathy-associated genes (n= 327) in COPD whole lung tissues: GSE76925** 19](#_Toc60660477)

[**Table S5. List of deregulated cilia/ciliopathy-associated genes (n= 396) in COPD whole lung tissues: GSE103174** 22](#_Toc60660478)

[**Table S6. List of deregulated cilia/ciliopathy-associated genes (n= 398) in COPD SAEC: GSE11784** 24](#_Toc60660479)

[**Table S7. List of deregulated cilia/ciliopathy-associated genes (n= 154) in COPD SAEC: GSE37147** 25](#_Toc60660480)

[**Table S8. List of deregulated cilia/ciliopathy-associated genes (n= 419) in COPD SAEC: GSE56341** 27](#_Toc60660481)

[**2. Supporting information Figures** 28](#_Toc60660482)

[**Figure S1. Localization and single-cell transcriptomic signatures of the main cilia hits in COPD whole lung tissues.** 28](#_Toc60660483)

[**Figure S2. Localization and single-cell transcriptomic signatures of the main cilia hits in COPD SAEC.** 29](#_Toc60660484)

[**Figure S3. Common cilia and ciliopathy-associated deregulated genes signature in whole lung and SAEC.** 30](#_Toc60660485)

1. **Additional information Tables**

## **Table S1. List of cilia/ciliopathy-associated genes in the cilia geneset (n=495).**

| **Gene Symbol** | **Gene ID** | **Cilia-associated genes** | **Ciliopathy-associated genes** |
| --- | --- | --- | --- |
| ABHD2 | 11057 |  |  |
| ADAM15 | 8751 |  |  |
| ADAM17 | 6868 |  |  |
| ADCY10 | 55811 |  |  |
| ADCY3 | 109 |  |  |
| ADCY5 | 111 |  |  |
| ADCY6 | 112 |  |  |
| ADGRV1 | 84059 |  |  |
| AGBL2 | 79841 |  |  |
| AGBL4 | 84871 |  |  |
| AGBL5 | 60509 |  |  |
| AGTPBP1 | 23287 |  |  |
| AHI1 | 54806 |  |  |
| AK7 | 122481 |  |  |
| AKAP14 | 158798 |  |  |
| AKAP4 | 8852 |  |  |
| ALG8 | 79053 |  |  |
| ALMS1 | 7840 |  |  |
| ANKMY2 | 57037 |  |  |
| ANKS6 | 203286 |  |  |
| ANXA1 | 301 |  |  |
| ARHGAP35 | 2909 |  |  |
| ARL13B | 200894 |  |  |
| ARL2BP | 23568 |  |  |
| ARL3 | 403 |  |  |
| ARL6 | 84100 |  |  |
| ARMC4 | 55130 |  |  |
| ARMC9 | 80210 |  |  |
| ARSG | 22901 |  |  |
| ATP2B4 | 493 |  |  |
| AURKA | 6790 |  |  |
| B9D1 | 27077 |  |  |
| B9D2 | 80776 |  |  |
| BBIP1 | 92482 |  |  |
| BBOF1 | 80127 |  |  |
| BBS1 | 582 |  |  |
| BBS10 | 79738 |  |  |
| BBS12 | 166379 |  |  |
| BBS2 | 583 |  |  |
| BBS4 | 585 |  |  |
| BBS5 | 129880 |  |  |
| BBS7 | 55212 |  |  |
| BBS9 | 27241 |  |  |
| C10orf90 | 118611 |  |  |
| C14orf104 | 55172 |  |  |
| C21ORF2 | - |  |  |
| C21orf59 | - |  |  |
| C21orf71 | - |  |  |
| C2CD3 | 26005 |  |  |
| C5orf30 | - |  |  |
| C5orf42 | - |  |  |
| C8orf37 | 157657 |  |  |
| CABCOCO1 | 219621 |  |  |
| CABS1 | 85438 |  |  |
| CABYR | 26256 |  |  |
| CALM1 | 801 |  |  |
| CALM2 | 805 |  |  |
| CALM3 | 808 |  |  |
| CATSPER1 | 117144 |  |  |
| CATSPER2 | 117155 |  |  |
| CATSPER3 | 347732 |  |  |
| CATSPER4 | 378807 |  |  |
| CATSPERD | 257062 |  |  |
| CATSPERE | 257044 |  |  |
| CATSPERZ | 25858 |  |  |
| CC2D2A | 57545 |  |  |
| CCDC103 | 388389 |  |  |
| CCDC11 | - |  |  |
| CCDC114 | 93233 |  |  |
| CCDC13 | 152206 |  |  |
| CCDC151 | 115948 |  |  |
| CCDC181 | 57821 |  |  |
| CCDC28B | 79140 |  |  |
| CCDC39 | 339829 |  |  |
| CCDC40 | 55036 |  |  |
| CCDC65 | 85478 |  |  |
| CCDC66 | 285331 |  |  |
| CCNO | 10309 |  |  |
| CCP110 | 9738 |  |  |
| CCSAP | 126731 |  |  |
| CCT2 | 10576 |  |  |
| CCT3 | 7203 |  |  |
| CCT4 | 10575 |  |  |
| CCT5 | 22948 |  |  |
| CCT8 | 10694 |  |  |
| CDH23 | 64072 |  |  |
| CDHR1 | 92211 |  |  |
| CDK10 | 8558 |  |  |
| CDK20 | 23552 |  |  |
| CELSR2 | 1952 |  |  |
| CENPF | 1063 |  |  |
| CENPJ | 55835 |  |  |
| CEP104 | 9731 |  |  |
| CEP120 | 153241 |  |  |
| CEP131 | 22994 |  |  |
| CEP162 | 22832 |  |  |
| CEP164 | 22897 |  |  |
| CEP19 | 84984 |  |  |
| CEP250 | 11190 |  |  |
| CEP290 | 80184 |  |  |
| CEP295NL | 100653515 |  |  |
| CEP41 | 95681 |  |  |
| CEP55 | 55165 |  |  |
| CEP78 | 84131 |  |  |
| CEP83 | 51134 |  |  |
| CEP89 | 84902 |  |  |
| CEP97 | 79598 |  |  |
| CETN1 | 1068 |  |  |
| CFAP100 | 348807 |  |  |
| CFAP126 | 257177 |  |  |
| CFAP157 | 286207 |  |  |
| CFAP161 | 161502 |  |  |
| CFAP20 | 29105 |  |  |
| CFAP206 | 154313 |  |  |
| CFAP221 | 200373 |  |  |
| CFAP298 | 56683 |  |  |
| CFAP299 | 255119 |  |  |
| CFAP300 | 85016 |  |  |
| CFAP36 | 112942 |  |  |
| CFAP410 | 755 |  |  |
| CFAP43 | 80217 |  |  |
| CFAP44 | 55779 |  |  |
| CFAP45 | 25790 |  |  |
| CFAP46 | 54777 |  |  |
| CFAP47 | 286464 |  |  |
| CFAP52 | 146845 |  |  |
| CFAP53 | 220136 |  |  |
| CFAP54 | 144535 |  |  |
| CFAP57 | 149465 |  |  |
| CFAP58 | 159686 |  |  |
| CFAP61 | 26074 |  |  |
| CFAP65 | 255101 |  |  |
| CFAP69 | 79846 |  |  |
| CFAP70 | 118491 |  |  |
| CFAP73 | 387885 |  |  |
| CFAP74 | 85452 |  |  |
| CFAP77 | 389799 |  |  |
| CFAP97 | 57587 |  |  |
| CFAP99 | 402160 |  |  |
| CFTR | 1080 |  |  |
| CIB2 | 10518 |  |  |
| CLRN1 (USH3A) | - |  |  |
| CLUAP1 | 23059 |  |  |
| CPLANE1 | 65250 |  |  |
| CPLANE2 | 79363 |  |  |
| CROCC | 9696 |  |  |
| CSPP1 | 79848 |  |  |
| CUL3 | 8452 |  |  |
| CYS1 | 192668 |  |  |
| DAAM1 | 23002 |  |  |
| DAW1 | 164781 |  |  |
| DCDC2 | 51473 |  |  |
| DCDC2C | 728597 |  |  |
| DCTN1 | 1639 |  |  |
| DDX59 | 83479 |  |  |
| DLD | 1738 |  |  |
| DNAAF1 | 123872 |  |  |
| DNAAF2 | 55172 |  |  |
| DNAAF3 | 352909 |  |  |
| DNAAF4 | 161582 |  |  |
| DNAAF5 | 54919 |  |  |
| DNAH1 | 25981 |  |  |
| DNAH10 | 196385 |  |  |
| DNAH11 | 8701 |  |  |
| DNAH12 | 201625 |  |  |
| DNAH14 | 127602 |  |  |
| DNAH17 | 8632 |  |  |
| DNAH2 | 146754 |  |  |
| DNAH3 | 55567 |  |  |
| DNAH5 | 1767 |  |  |
| DNAH6 | 1768 |  |  |
| DNAH7 | 56171 |  |  |
| DNAH8 | 1769 |  |  |
| DNAH9 | 1770 |  |  |
| DNAI1 | 27019 |  |  |
| DNAI2 | 64446 |  |  |
| DNAJB13 | 374407 |  |  |
| DNAL1 | 83544 |  |  |
| DNAL4 | 10126 |  |  |
| DRC1 | 92749 |  |  |
| DRC3 | 83450 |  |  |
| DRC7 | 84229 |  |  |
| DYNC2H1 | 79659 |  |  |
| DYNC2LI1 | 51626 |  |  |
| DYX1C1 | 161582 |  |  |
| DZIP1 | 22873 |  |  |
| DZIP1L | 199221 |  |  |
| EFCAB2 | 84288 |  |  |
| EFCAB7 | 84455 |  |  |
| EFCAB9 | 285588 |  |  |
| EGLN2 | 112398 |  |  |
| EHD1 | 10938 |  |  |
| EHD3 | 30845 |  |  |
| ELMOD3 | 84173 |  |  |
| ENKUR | 219670 |  |  |
| ENTR1 | 10807 |  |  |
| EVC | 2121 |  |  |
| EVC2 | 132884 |  |  |
| EXOC4 | 60412 |  |  |
| EXOC8 | 149371 |  |  |
| EYS | 346007 |  |  |
| FAM161A | 84140 |  |  |
| FAT4 | 79633 |  |  |
| FBF1 | 85302 |  |  |
| FBXL13 | 222235 |  |  |
| FHDC1 | 85462 |  |  |
| FLACC1 | 130540 |  |  |
| FOPNL | 123811 |  |  |
| FOXJ1 | 2302 |  |  |
| FSCB | 84075 |  |  |
| FUZ | 80199 |  |  |
| GALNT11 | 63917 |  |  |
| GANAB | 23193 |  |  |
| GAS2L2 | 246176 |  |  |
| GAS8 | 2622 |  |  |
| GLI2 | 2736 |  |  |
| GLI3 | 2737 |  |  |
| GLIS2 | 84662 |  |  |
| GPR157 | 80045 |  |  |
| GPR161 | 23432 |  |  |
| GPR37L1 | 9283 |  |  |
| GRXCR1 | 389207 |  |  |
| HARS | - |  |  |
| HSPB11 | 51668 |  |  |
| HYDIN | 54768 |  |  |
| HYLS1 | 219844 |  |  |
| ICK | 22858 |  |  |
| IFT122 | 55764 |  |  |
| IFT140 | 9742 |  |  |
| IFT172 | 26160 |  |  |
| IFT20 | 90410 |  |  |
| IFT22 | 64792 |  |  |
| IFT27 | 11020 |  |  |
| IFT43 | 112752 |  |  |
| IFT46 | 56912 |  |  |
| IFT52 | 51098 |  |  |
| IFT57 | 55081 |  |  |
| IFT74 | 80173 |  |  |
| IFT80 | 57560 |  |  |
| IFT81 | 28981 |  |  |
| IFT88 | 8100 |  |  |
| INPP5E | 56623 |  |  |
| INTU | 27152 |  |  |
| INVS | 27130 |  |  |
| IQCA1 | 79781 |  |  |
| IQCB1 | 9657 |  |  |
| IQCD | 115811 |  |  |
| IQCE | 23288 |  |  |
| IQCG | 84223 |  |  |
| IQUB | 154865 |  |  |
| JHY | 79864 |  |  |
| KATNB1 | 10300 |  |  |
| KIAA0556 | 23247 |  |  |
| KIAA0586 | 9786 |  |  |
| KIAA0753 | 9851 |  |  |
| KIF14 | 9928 |  |  |
| KIF19 | 124602 |  |  |
| KIF24 | 347240 |  |  |
| KIF27 | 55582 |  |  |
| KIF3A | 11127 |  |  |
| KIF7 | 374654 |  |  |
| KIFC1 | 3833 |  |  |
| LCA5 | 167691 |  |  |
| LRGUK | 136332 |  |  |
| LRRC56 | 115399 |  |  |
| LRRC6 | 23639 |  |  |
| LRRK2 | 120892 |  |  |
| LYZL4 | 131375 |  |  |
| LYZL6 | 57151 |  |  |
| LZTFL1 | 54585 |  |  |
| MAATS1 | 89876 |  |  |
| MAK | 4117 |  |  |
| MAPK9 | 5601 |  |  |
| MAPKAP1 | 79109 |  |  |
| MAPKBP1 | 23005 |  |  |
| MCIDAS | 345643 |  |  |
| MKKS | 8195 |  |  |
| MKS1 | 54903 |  |  |
| MNS1 | 55329 |  |  |
| MROH2B | 133558 |  |  |
| MTCL1 | 23255 |  |  |
| MXRA8 | 54587 |  |  |
| MYO1D | 4642 |  |  |
| MYO7A | 4647 |  |  |
| MYOC | 4653 |  |  |
| NEK1 | 4750 |  |  |
| NEK2 | 4751 |  |  |
| NEK6 | 10783 |  |  |
| NEK8 | 284086 |  |  |
| NME8 | 51314 |  |  |
| NME9 | 347736 |  |  |
| NOTCH1 | 4851 |  |  |
| NOTO | 344022 |  |  |
| NPHP1 | 4867 |  |  |
| NPHP3 | 27031 |  |  |
| NPHP4 | 261734 |  |  |
| NUBP1 | 4682 |  |  |
| NUBP2 | 10101 |  |  |
| OCRL | 4952 |  |  |
| ODF2 | 4957 |  |  |
| OFD1 | 8481 |  |  |
| PACS1 | 55690 |  |  |
| PCARE | 388939 |  |  |
| PCDH15 | 65217 |  |  |
| PCM1 | 5108 |  |  |
| PDC | 5132 |  |  |
| PDE1C | 5137 |  |  |
| PDE4C | 5143 |  |  |
| PDE6D | 5147 |  |  |
| PDZD7 | 79955 |  |  |
| PEX6 | 5190 |  |  |
| PIBF1 | 10464 |  |  |
| PIFO | 128344 |  |  |
| PIH1D3 | 139212 |  |  |
| PKD1 | 5310 |  |  |
| PKD1L1 | 168507 |  |  |
| PKD2 | 5311 |  |  |
| PKD2L1 | 9033 |  |  |
| PKHD1 | 5314 |  |  |
| PMFBP1 | 83449 |  |  |
| POC1B | 282809 |  |  |
| PPEF2 | 5470 |  |  |
| PRICKLE3 | 4007 |  |  |
| PRKACA | 5566 |  |  |
| PRKCSH | 5589 |  |  |
| PROM1 | 8842 |  |  |
| PROM2 | 150696 |  |  |
| PRPF3 | 9129 |  |  |
| PRPF31 | 26121 |  |  |
| PRPF4 | 9128 |  |  |
| PRPF6 | 24148 |  |  |
| PRPF8 | 10594 |  |  |
| PTPDC1 | 138639 |  |  |
| PTPN23 | 25930 |  |  |
| QRICH2 | 84074 |  |  |
| RAB10 | 10890 |  |  |
| RAB6A | 5870 |  |  |
| RAB8A | 4218 |  |  |
| RABL2A | 11159 |  |  |
| RABL2B | 11158 |  |  |
| RFX3 | 5991 |  |  |
| RHO | 6010 |  |  |
| RICTOR | 253260 |  |  |
| RILPL1 | 353116 |  |  |
| RILPL2 | 196383 |  |  |
| RO60 | 6738 |  |  |
| ROPN1 | 54763 |  |  |
| ROPN1B | 152015 |  |  |
| ROPN1L | 83853 |  |  |
| RP1 | 6101 |  |  |
| RP1L1 | 94137 |  |  |
| RP2 | 6102 |  |  |
| RPGR | 6103 |  |  |
| RPGRIP1 | 57096 |  |  |
| RPGRIP1L | 23322 |  |  |
| RPTOR | 57521 |  |  |
| RSPH1 | 89765 |  |  |
| RSPH3 | 83861 |  |  |
| RSPH4A | 345895 |  |  |
| RSPH9 | 221421 |  |  |
| RTTN | 25914 |  |  |
| SANS | - |  |  |
| SAXO1 | 158297 |  |  |
| SCLT1 | 132320 |  |  |
| SCNN1A | 6337 |  |  |
| SCNN1B | 6338 |  |  |
| SCNN1G | 6340 |  |  |
| SDCCAG8 | 10806 |  |  |
| SEC63 | 11231 |  |  |
| SEPTIN12 | 124404 |  |  |
| SEPTIN2 | 4735 |  |  |
| SEPTIN4 | 5414 |  |  |
| SEPTIN6 | 23157 |  |  |
| SEPTIN7 | 989 |  |  |
| SLC25A31 | 83447 |  |  |
| SLC9B1 | 150159 |  |  |
| SLC9B2 | 133308 |  |  |
| SLC9C1 | 285335 |  |  |
| SMO | 6608 |  |  |
| SNAP29 | 9342 |  |  |
| SNRNP200 | 23020 |  |  |
| SNTN | 132203 |  |  |
| SORD | 6652 |  |  |
| SPACA9 | 11092 |  |  |
| SPAG1 | 6674 |  |  |
| SPAG16 | 79582 |  |  |
| SPAG17 | 200162 |  |  |
| SPAG4 | 6676 |  |  |
| SPAG6 | 9576 |  |  |
| SPAST | 6683 |  |  |
| SPATA6 | 54558 |  |  |
| SPATA7 | 55812 |  |  |
| SPEF1 | 25876 |  |  |
| SSX2IP | 117178 |  |  |
| STARD10 | 10809 |  |  |
| STK36 | 27148 |  |  |
| STRC | 161497 |  |  |
| SUFU | 51684 |  |  |
| TAPT1 | 202018 |  |  |
| TAS2R4 | 50832 |  |  |
| TAS2R43 | 259289 |  |  |
| TAS2R46 | 259292 |  |  |
| TBC1D32 | 221322 |  |  |
| TBCCD1 | 55171 |  |  |
| TBX6 | 6911 |  |  |
| TCP1 | 6950 |  |  |
| TCP11 | 6954 |  |  |
| TCTE1 | 202500 |  |  |
| TCTEX1D2 | 255758 |  |  |
| TCTN1 | 79600 |  |  |
| TCTN2 | 79867 |  |  |
| TCTN3 | 26123 |  |  |
| TEKT1 | 83659 |  |  |
| TEKT2 | 27285 |  |  |
| TEKT3 | 64518 |  |  |
| TEKT4 | 150483 |  |  |
| TEKT5 | 146279 |  |  |
| TMEM107 | 84314 |  |  |
| TMEM138 | 51524 |  |  |
| TMEM17 | 200728 |  |  |
| TMEM216 | 51259 |  |  |
| TMEM231 | 79583 |  |  |
| TMEM237 | 65062 |  |  |
| TMEM67 | 91147 |  |  |
| TOGARAM1 | 23116 |  |  |
| TOPORS | 10210 |  |  |
| TPGS1 | 91978 |  |  |
| TPPP2 | 122664 |  |  |
| TRAF3IP1 | 26146 |  |  |
| TRIM32 | 22954 |  |  |
| TRPV4 | 59341 |  |  |
| TSSK1B | 83942 |  |  |
| TSSK4 | 283629 |  |  |
| TTBK2 | 146057 |  |  |
| TTC21B | 79809 |  |  |
| TTC23 | 64927 |  |  |
| TTC25 | 83538 |  |  |
| TTC26 | 79989 |  |  |
| TTC30A | 92104 |  |  |
| TTC30B | 150737 |  |  |
| TTC8 | 123016 |  |  |
| TTLL11 | 158135 |  |  |
| TTLL3 | 26140 |  |  |
| TTLL4 | 9654 |  |  |
| TTLL5 | 23093 |  |  |
| TTLL6 | 284076 |  |  |
| TTLL7 | 79739 |  |  |
| TTLL8 | 164714 |  |  |
| TTLL9 | 164395 |  |  |
| TUBA1A | 7846 |  |  |
| TUBA1B | 10376 |  |  |
| TUBA1C | 84790 |  |  |
| TUBA3C | 7278 |  |  |
| TUBA3D | 113457 |  |  |
| TUBA3E | 112714 |  |  |
| TUBA4A | 7277 |  |  |
| TUBA4B | 80086 |  |  |
| TUBA8 | 51807 |  |  |
| TUBAL3 | 79861 |  |  |
| TUBB | 203068 |  |  |
| TUBB1 | 81027 |  |  |
| TUBB2A | 7280 |  |  |
| TUBB2B | 347733 |  |  |
| TUBB3 | 10381 |  |  |
| TUBB4A | 10382 |  |  |
| TUBB4B | 10383 |  |  |
| TUBB6 | 84617 |  |  |
| TUBB8 | 347688 |  |  |
| TUBB8B | 260334 |  |  |
| TUBG1 | 7283 |  |  |
| TULP1 | 7287 |  |  |
| TULP3 | 7289 |  |  |
| UBXN10 | 127733 |  |  |
| UMOD | 7369 |  |  |
| UNC119B | 84747 |  |  |
| USH1C | 10083 |  |  |
| USH1E | 7396 |  |  |
| USH1H | 100271837 |  |  |
| USH1K | 101180907 |  |  |
| USH2A | 7399 |  |  |
| VHL | 7428 |  |  |
| WDPCP | 51057 |  |  |
| WDR11 | 55717 |  |  |
| WDR19 | 57728 |  |  |
| WDR34 | 89891 |  |  |
| WDR35 | 57539 |  |  |
| WDR60 | 55112 |  |  |
| WDR62 | 284403 |  |  |
| WDR66 | 144406 |  |  |
| WHRN | 25861 |  |  |
| WTIP | 126374 |  |  |
| XPNPEP2 | 7512 |  |  |
| XPNPEP3 | 63929 |  |  |
| ZMYND10 | 51364 |  |  |
| ZNF423 | 23090 |  |  |

## **Table S2. List of deregulated cilia/ciliopathy-associated genes (n=278) in COPD whole lung tissues: GSE47460**

|  | **non-COPD** | | **COPD** | |  |  |  |  |
| --- | --- | --- | --- | --- | --- | --- | --- | --- |
| **Genes Ids** | **mean** | **sd** | **mean** | **sd** | **FC** | **log2FC** | **p value** | **FDR p value** |
| ANKS6 | 12.1003 | 0.1962 | 12.0374 | 0.2189 | 1.0052 | 0.0075 | 0.0265 | 0.1391 |
| ARL2BP | 12.3083 | 0.1628 | 12.2423 | 0.2015 | 1.0054 | 0.0078 | 0.0091 | 0.0651 |
| ATP2B4 | 5.5570 | 0.3573 | 5.3107 | 0.3878 | 1.0464 | 0.0654 | <10-4 | 0.0003 |
| B9D2 | 10.3499 | 0.3917 | 10.1860 | 0.3708 | 1.0161 | 0.0230 | 0.0014 | 0.0182 |
| BBS4 | 10.8525 | 0.2858 | 10.7343 | 0.3466 | 1.0110 | 0.0158 | 0.0070 | 0.0555 |
| BBS9 | 9.8464 | 0.2443 | 9.7487 | 0.2701 | 1.0100 | 0.0144 | 0.0055 | 0.0475 |
| C21ORF2 | 8.7746 | 0.3374 | 8.6316 | 0.2814 | 1.0166 | 0.0237 | 0.0005 | 0.0104 |
| CABYR | 5.1007 | 0.8782 | 5.3605 | 0.8771 | 0.9515 | -0.0717 | 0.0278 | 0.1406 |
| CALM3 | 11.0325 | 0.4353 | 11.1980 | 0.3182 | 0.9852 | -0.0215 | 0.0009 | 0.0165 |
| CATSPER1 | 7.3683 | 0.5476 | 7.5926 | 0.6271 | 0.9705 | -0.0432 | 0.0055 | 0.0475 |
| CC2D2A | 7.6555 | 0.5183 | 7.5000 | 0.5440 | 1.0207 | 0.0296 | 0.0306 | 0.1464 |
| CCT8 | 12.5140 | 0.3775 | 12.6370 | 0.2223 | 0.9903 | -0.0141 | 0.0018 | 0.0206 |
| CDHR1 | 4.1163 | 0.8000 | 4.3403 | 0.8084 | 0.9484 | -0.0765 | 0.0386 | 0.1602 |
| CDK20 | 9.6527 | 0.3757 | 9.5466 | 0.3860 | 1.0111 | 0.0159 | 0.0389 | 0.1602 |
| CEP164 | 9.0146 | 0.2203 | 8.9309 | 0.2391 | 1.0094 | 0.0135 | 0.0075 | 0.0563 |
| CEP97 | 4.3660 | 0.7107 | 4.5616 | 0.6688 | 0.9571 | -0.0632 | 0.0338 | 0.1492 |
| CUL3 | 6.6474 | 0.5353 | 6.4093 | 0.4276 | 1.0371 | 0.0526 | 0.0002 | 0.0057 |
| DAAM1 | 9.3436 | 0.5282 | 9.2161 | 0.3840 | 1.0138 | 0.0198 | 0.0333 | 0.1492 |
| DNAH17 | 4.7310 | 0.6599 | 4.3507 | 0.7748 | 1.0874 | 0.1209 | 0.0001 | 0.0054 |
| DNAH8 | 2.1837 | 0.5730 | 2.3933 | 0.8157 | 0.9124 | -0.1322 | 0.0333 | 0.1492 |
| DNAL4 | 8.5902 | 0.3102 | 8.4856 | 0.3081 | 1.0123 | 0.0177 | 0.0120 | 0.0744 |
| DZIP1 | 8.5046 | 0.4499 | 8.6315 | 0.3960 | 0.9853 | -0.0214 | 0.0240 | 0.1306 |
| EHD1 | 13.5451 | 0.3313 | 13.4191 | 0.3926 | 1.0094 | 0.0135 | 0.0115 | 0.0735 |
| EHD3 | 8.3630 | 0.5959 | 8.1851 | 0.5377 | 1.0217 | 0.0310 | 0.0185 | 0.1052 |
| FHDC1 | 7.5288 | 0.5475 | 7.1992 | 0.7556 | 1.0458 | 0.0646 | 0.0004 | 0.0088 |
| GLI2 | 7.5184 | 0.4596 | 7.7042 | 0.5133 | 0.9759 | -0.0352 | 0.0053 | 0.0475 |
| HARS | 10.9850 | 0.1705 | 11.0362 | 0.1331 | 0.9954 | -0.0067 | 0.0106 | 0.0721 |
| ICK | 10.3705 | 0.4943 | 10.4705 | 0.2658 | 0.9904 | -0.0139 | 0.0448 | 0.1778 |
| IFT122 | 10.0491 | 0.2895 | 9.9452 | 0.2768 | 1.0105 | 0.0150 | 0.0062 | 0.0510 |
| IFT140 | 9.3394 | 0.3911 | 9.1326 | 0.4172 | 1.0226 | 0.0323 | 0.0002 | 0.0057 |
| INVS | 8.1750 | 0.2518 | 8.0799 | 0.2688 | 1.0118 | 0.0169 | 0.0072 | 0.0557 |
| IQCB1 | 10.3479 | 0.4010 | 10.4772 | 0.3330 | 0.9877 | -0.0179 | 0.0079 | 0.0574 |
| IQCD | 9.2539 | 1.1201 | 8.9215 | 1.2522 | 1.0373 | 0.0528 | 0.0399 | 0.1608 |
| IQCE | 11.7634 | 0.3633 | 11.6076 | 0.3439 | 1.0134 | 0.0192 | 0.0011 | 0.0165 |
| KIAA0556 | 5.5563 | 0.4301 | 5.3115 | 0.6083 | 1.0461 | 0.0650 | 0.0009 | 0.0165 |
| KIAA0753 | 9.9121 | 0.2714 | 9.8184 | 0.2387 | 1.0095 | 0.0137 | 0.0058 | 0.0491 |
| KIFC1 | 6.0467 | 0.6498 | 5.8511 | 0.6802 | 1.0334 | 0.0475 | 0.0296 | 0.1445 |
| LRRC56 | 10.1362 | 0.7349 | 9.9163 | 0.7562 | 1.0222 | 0.0316 | 0.0289 | 0.1436 |
| MYO7A | 4.0701 | 1.2065 | 4.4640 | 1.2093 | 0.9118 | -0.1333 | 0.0155 | 0.0919 |
| MYOC | 10.0159 | 1.3627 | 9.4814 | 1.3267 | 1.0564 | 0.0791 | 0.0032 | 0.0319 |
| NEK6 | 10.2409 | 0.4805 | 10.5674 | 0.4728 | 0.9691 | -0.0453 | <10-4 | 0.0002 |
| NUBP2 | 10.1541 | 0.2257 | 10.0800 | 0.2095 | 1.0074 | 0.0106 | 0.0109 | 0.0722 |
| PDE6D | 9.0126 | 0.1635 | 9.0757 | 0.1498 | 0.9930 | -0.0101 | 0.0026 | 0.0282 |
| PRPF3 | 8.8859 | 0.1740 | 8.8291 | 0.1837 | 1.0064 | 0.0093 | 0.0190 | 0.1055 |
| PRPF8 | 12.1977 | 0.2077 | 12.1423 | 0.1800 | 1.0046 | 0.0066 | 0.0311 | 0.1464 |
| PTPDC1 | 8.8577 | 0.2719 | 8.7293 | 0.2538 | 1.0147 | 0.0211 | 0.0003 | 0.0074 |
| QRICH2 | 4.0463 | 0.7412 | 3.6814 | 0.7954 | 1.0991 | 0.1364 | 0.0005 | 0.0104 |
| RFX3 | 7.6931 | 0.4658 | 7.5332 | 0.4964 | 1.0212 | 0.0303 | 0.0144 | 0.0870 |
| RILPL1 | 10.1056 | 0.2588 | 10.0021 | 0.2352 | 1.0104 | 0.0149 | 0.0018 | 0.0206 |
| SCNN1A | 9.6000 | 0.3767 | 9.3415 | 0.6907 | 1.0277 | 0.0394 | 0.0012 | 0.0169 |
| SPAG4 | 7.2741 | 0.7426 | 7.7360 | 0.9970 | 0.9403 | -0.0888 | 0.0002 | 0.0057 |
| STARD10 | 10.4975 | 0.3534 | 10.4018 | 0.3619 | 1.0092 | 0.0132 | 0.0472 | 0.1847 |
| SUFU | 7.1370 | 0.4373 | 7.2861 | 0.4737 | 0.9795 | -0.0298 | 0.0161 | 0.0931 |
| TAPT1 | 10.5530 | 0.2652 | 10.4004 | 0.2606 | 1.0147 | 0.0210 | <10-4 | 0.0014 |
| TCTN2 | 6.3882 | 0.5430 | 6.1963 | 0.5772 | 1.0310 | 0.0440 | 0.0116 | 0.0735 |
| TEKT2 | 9.2027 | 1.8405 | 8.6468 | 2.0665 | 1.0643 | 0.0899 | 0.0371 | 0.1587 |
| TMEM138 | 9.3330 | 0.2836 | 9.4819 | 0.2284 | 0.9843 | -0.0228 | <10-4 | 0.0013 |
| TMEM237 | 8.5652 | 0.3826 | 8.4480 | 0.3952 | 1.0139 | 0.0199 | 0.0257 | 0.1375 |
| TTC30B | 7.5809 | 0.5206 | 7.3525 | 0.5556 | 1.0311 | 0.0441 | 0.0019 | 0.0206 |
| TTLL11 | 6.6289 | 0.2934 | 6.5172 | 0.3421 | 1.0171 | 0.0245 | 0.0106 | 0.0721 |
| TTLL5 | 7.8936 | 0.2544 | 7.7873 | 0.2913 | 1.0137 | 0.0196 | 0.0046 | 0.0438 |
| TTLL6 | 4.8833 | 1.2673 | 4.2581 | 1.4865 | 1.1468 | 0.1977 | 0.0010 | 0.0165 |
| TUBA3D | 8.5678 | 0.4833 | 8.7336 | 0.6282 | 0.9810 | -0.0277 | 0.0326 | 0.1492 |
| TUBB3 | 6.9171 | 1.4125 | 7.7611 | 1.5274 | 0.8913 | -0.1661 | <10-4 | 0.0017 |
| TUBB4A | 3.8819 | 0.5471 | 4.1384 | 0.6038 | 0.9380 | -0.0923 | 0.0012 | 0.0169 |
| TUBB6 | 15.1883 | 0.3734 | 15.0108 | 0.4338 | 1.0118 | 0.0170 | 0.0014 | 0.0182 |
| UNC119B | 10.4551 | 0.3678 | 10.3530 | 0.3682 | 1.0099 | 0.0142 | 0.0392 | 0.1602 |
| WDPCP | 6.9623 | 0.2760 | 6.8760 | 0.3197 | 1.0126 | 0.0180 | 0.0344 | 0.1496 |
| WDR11 | 9.4065 | 0.3054 | 9.5060 | 0.2077 | 0.9895 | -0.0152 | 0.0032 | 0.0319 |
| WDR34 | 10.9343 | 0.3610 | 10.7316 | 0.3937 | 1.0189 | 0.0270 | 0.0001 | 0.0044 |
| XPNPEP3 | 5.4186 | 0.3841 | 5.2847 | 0.4882 | 1.0253 | 0.0361 | 0.0274 | 0.1406 |

## **Table S3. List of deregulated cilia/ciliopathy-associated genes (n=378) in COPD whole lung tissues: GSE57148**

|  | **non-COPD** | | **COPD** | |  |  |  |  |
| --- | --- | --- | --- | --- | --- | --- | --- | --- |
| **Genes Ids** | **mean** | **sd** | **mean** | **sd** | **FC** | **log2FC** | **p value** | **FDR p value** |
| ABHD2 | 30.5919 | 8.0890 | 39.5751 | 10.8610 | 0.7730 | -0.3714 | <10-4 | <10-4 |
| ADAM15 | 24.0317 | 6.1987 | 21.3341 | 5.3843 | 1.1264 | 0.1718 | 0.0016 | 0.0063 |
| ADAM17 | 16.8920 | 4.9133 | 22.4564 | 7.2946 | 0.7522 | -0.4108 | <10-4 | <10-4 |
| ADCY3 | 8.7903 | 3.2396 | 9.8992 | 3.7982 | 0.8880 | -0.1714 | 0.0327 | 0.0847 |
| ADCY6 | 10.3504 | 2.2062 | 9.6101 | 2.0268 | 1.0770 | 0.1071 | 0.0172 | 0.0512 |
| AGTPBP1 | 9.3039 | 2.2944 | 10.1181 | 2.5985 | 0.9195 | -0.1210 | 0.0240 | 0.0691 |
| ALMS1 | 1.3214 | 0.3600 | 1.7885 | 0.7874 | 0.7388 | -0.4367 | <10-4 | <10-4 |
| ARHGAP35 | 11.9836 | 2.9492 | 14.0446 | 3.0463 | 0.8533 | -0.2290 | <10-4 | <10-4 |
| ARL13B | 8.1691 | 2.4841 | 8.9336 | 2.4753 | 0.9144 | -0.1291 | 0.0355 | 0.0895 |
| ARL6 | 3.6996 | 1.3198 | 4.5837 | 1.4655 | 0.8071 | -0.3091 | <10-4 | 0.0002 |
| ARMC9 | 3.6936 | 1.0474 | 4.2841 | 1.2863 | 0.8622 | -0.2140 | 0.0007 | 0.0033 |
| ARSG | 1.6158 | 0.4185 | 1.9884 | 0.4841 | 0.8126 | -0.2993 | <10-4 | <10-4 |
| ATP2B4 | 25.0510 | 5.4541 | 32.7335 | 8.3140 | 0.7653 | -0.3859 | <10-4 | <10-4 |
| B9D2 | 5.1629 | 1.9692 | 4.1508 | 2.3197 | 1.2438 | 0.3148 | 0.0015 | 0.0061 |
| BBS7 | 4.8148 | 0.9452 | 5.5685 | 1.1881 | 0.8647 | -0.2098 | <10-4 | <10-4 |
| BBS9 | 3.5166 | 0.7929 | 3.8510 | 1.0107 | 0.9132 | -0.1311 | 0.0127 | 0.0394 |
| C21ORF2 | 9.1685 | 3.0504 | 6.7629 | 2.5851 | 1.3557 | 0.4391 | <10-4 | <10-4 |
| C2CD3 | 2.3227 | 0.6927 | 3.5163 | 1.1072 | 0.6605 | -0.5983 | <10-4 | <10-4 |
| C5orf42 | 1.5029 | 0.3360 | 2.0037 | 0.8632 | 0.7500 | -0.4149 | <10-4 | <10-4 |
| C8orf37 | 1.5830 | 0.4698 | 1.7681 | 0.6450 | 0.8953 | -0.1596 | 0.0262 | 0.0713 |
| CALM1 | 117.2466 | 15.8381 | 122.5940 | 18.1369 | 0.9564 | -0.0643 | 0.0327 | 0.0847 |
| CALM3 | 72.6925 | 17.9571 | 78.6754 | 22.7636 | 0.9240 | -0.1141 | 0.0474 | 0.1127 |
| CATSPER1 | 0.6332 | 0.3210 | 0.5078 | 0.2627 | 1.2469 | 0.3183 | 0.0036 | 0.0130 |
| CC2D2A | 5.9470 | 2.1809 | 6.8678 | 3.8340 | 0.8659 | -0.2077 | 0.0460 | 0.1100 |
| CCDC28B | 3.5123 | 1.4561 | 2.6894 | 1.2182 | 1.3059 | 0.3851 | <10-4 | 0.0002 |
| CCT3 | 39.4539 | 6.2935 | 37.2164 | 4.8549 | 1.0601 | 0.0842 | 0.0066 | 0.0222 |
| CCT4 | 35.8322 | 4.8271 | 33.1252 | 3.6278 | 1.0817 | 0.1133 | <10-4 | 0.0001 |
| CDH23 | 3.6796 | 1.2999 | 4.2634 | 2.1543 | 0.8631 | -0.2125 | 0.0266 | 0.0719 |
| CDK10 | 22.9142 | 8.7551 | 16.3024 | 5.5538 | 1.4056 | 0.4912 | <10-4 | <10-4 |
| CELSR2 | 1.2036 | 0.3586 | 1.3749 | 0.4680 | 0.8754 | -0.1920 | 0.0055 | 0.0190 |
| CENPJ | 1.7863 | 0.3377 | 2.1109 | 0.4875 | 0.8462 | -0.2409 | <10-4 | <10-4 |
| CEP104 | 6.1707 | 1.1647 | 7.3822 | 1.3390 | 0.8359 | -0.2586 | <10-4 | <10-4 |
| CEP250 | 3.4011 | 0.7807 | 3.8881 | 0.9170 | 0.8748 | -0.1931 | 0.0001 | 0.0007 |
| CEP290 | 3.0614 | 0.7325 | 3.5286 | 1.3001 | 0.8676 | -0.2049 | 0.0029 | 0.0108 |
| CEP78 | 2.7893 | 0.6126 | 2.9819 | 0.6088 | 0.9354 | -0.0963 | 0.0316 | 0.0829 |
| CEP89 | 3.7181 | 0.8347 | 4.3652 | 0.9782 | 0.8518 | -0.2315 | <10-4 | <10-4 |
| CEP97 | 1.1510 | 0.4383 | 1.8052 | 0.9591 | 0.6376 | -0.6492 | <10-4 | <10-4 |
| CFTR | 3.6526 | 2.0690 | 4.7872 | 2.9460 | 0.7630 | -0.3903 | 0.0027 | 0.0101 |
| CUL3 | 8.8210 | 1.7244 | 9.6473 | 1.8007 | 0.9143 | -0.1292 | 0.0015 | 0.0061 |
| CYS1 | 4.9103 | 2.0319 | 3.9911 | 1.5916 | 1.2303 | 0.2990 | 0.0006 | 0.0031 |
| DAAM1 | 5.7383 | 2.0857 | 8.0926 | 2.7631 | 0.7091 | -0.4960 | <10-4 | <10-4 |
| DCTN1 | 32.6944 | 5.7258 | 35.2864 | 4.7356 | 0.9265 | -0.1101 | 0.0008 | 0.0037 |
| DDX59 | 9.8873 | 1.6546 | 11.4927 | 1.9274 | 0.8603 | -0.2171 | <10-4 | <10-4 |
| DNAH14 | 1.5052 | 0.6246 | 1.8586 | 0.5472 | 0.8098 | -0.3043 | 0.0001 | 0.0003 |
| DNAL1 | 3.6230 | 1.2519 | 4.2993 | 2.0616 | 0.8427 | -0.2469 | 0.0076 | 0.0250 |
| DNAL4 | 11.5412 | 3.5338 | 9.7127 | 4.9438 | 1.1883 | 0.2488 | 0.0041 | 0.0145 |
| DYNC2H1 | 1.4826 | 0.6963 | 1.9240 | 1.3967 | 0.7706 | -0.3759 | 0.0072 | 0.0241 |
| DZIP1 | 2.1631 | 0.6214 | 2.5180 | 0.8376 | 0.8591 | -0.2191 | 0.0012 | 0.0050 |
| EGLN2 | 36.2927 | 8.9284 | 28.9033 | 7.3177 | 1.2557 | 0.3284 | <10-4 | <10-4 |
| ELMOD3 | 7.2635 | 1.7950 | 6.2544 | 1.2274 | 1.1614 | 0.2158 | <10-4 | 0.0001 |
| EVC | 4.4883 | 1.5093 | 6.8045 | 2.1310 | 0.6596 | -0.6003 | <10-4 | <10-4 |
| EVC2 | 1.6703 | 0.4815 | 1.8698 | 0.4173 | 0.8933 | -0.1628 | 0.0026 | 0.0100 |
| EXOC4 | 14.6975 | 3.5159 | 19.6972 | 5.4375 | 0.7462 | -0.4224 | <10-4 | <10-4 |
| EXOC8 | 5.7544 | 1.2046 | 6.6185 | 1.4087 | 0.8695 | -0.2018 | <10-4 | 0.0001 |
| FAT4 | 2.3718 | 0.8064 | 3.0333 | 1.0323 | 0.7819 | -0.3549 | <10-4 | <10-4 |
| FOPNL | 15.6571 | 2.4333 | 14.6141 | 2.0016 | 1.0714 | 0.0995 | 0.0015 | 0.0060 |
| FUZ | 7.7489 | 3.7131 | 6.3226 | 4.7890 | 1.2256 | 0.2935 | 0.0240 | 0.0691 |
| GALNT11 | 19.7130 | 2.4165 | 20.4982 | 2.3949 | 0.9617 | -0.0563 | 0.0261 | 0.0713 |
| GANAB | 53.7218 | 7.3295 | 57.5425 | 7.7967 | 0.9336 | -0.0991 | 0.0007 | 0.0032 |
| GLI2 | 1.2154 | 0.5681 | 1.3988 | 0.6361 | 0.8689 | -0.2028 | 0.0384 | 0.0962 |
| GLI3 | 2.4169 | 0.6843 | 2.9523 | 0.8601 | 0.8187 | -0.2887 | <10-4 | <10-4 |
| GLIS2 | 11.6667 | 3.3857 | 10.5872 | 3.9091 | 1.1020 | 0.1401 | 0.0445 | 0.1086 |
| GPR157 | 4.2139 | 1.6683 | 5.3185 | 1.7057 | 0.7923 | -0.3358 | <10-4 | 0.0001 |
| GPR161 | 1.9953 | 0.5910 | 2.6402 | 0.7821 | 0.7558 | -0.4040 | <10-4 | <10-4 |
| HARS | 20.5201 | 3.3753 | 18.8295 | 2.1343 | 1.0898 | 0.1240 | 0.0001 | 0.0003 |
| HSPB11 | 52.7790 | 12.5575 | 48.8147 | 11.3688 | 1.0812 | 0.1126 | 0.0239 | 0.0691 |
| HYDIN | 0.9759 | 1.1165 | 1.7769 | 2.8384 | 0.5492 | -0.8645 | 0.0127 | 0.0394 |
| HYLS1 | 1.8782 | 0.5596 | 1.6031 | 0.3720 | 1.1716 | 0.2284 | 0.0001 | 0.0005 |
| ICK | 4.4446 | 0.9636 | 4.9091 | 1.1246 | 0.9054 | -0.1434 | 0.0027 | 0.0101 |
| IFT27 | 9.0092 | 4.8585 | 7.3109 | 5.3946 | 1.2323 | 0.3014 | 0.0245 | 0.0691 |
| IFT43 | 32.5502 | 9.2674 | 22.7194 | 10.8394 | 1.4327 | 0.5187 | <10-4 | <10-4 |
| IFT52 | 15.1567 | 2.7396 | 16.1329 | 3.3572 | 0.9395 | -0.0901 | 0.0305 | 0.0806 |
| INPP5E | 5.6879 | 1.6718 | 5.1620 | 1.9454 | 1.1019 | 0.1400 | 0.0485 | 0.1146 |
| INVS | 3.2473 | 0.6698 | 4.3006 | 0.9727 | 0.7551 | -0.4053 | <10-4 | <10-4 |
| KIAA0556 | 4.5951 | 1.5707 | 5.2860 | 2.1383 | 0.8693 | -0.2021 | 0.0127 | 0.0394 |
| KIAA0586 | 2.1908 | 0.4997 | 2.6594 | 0.5972 | 0.8238 | -0.2797 | <10-4 | <10-4 |
| KIF27 | 1.3251 | 0.5541 | 1.9199 | 1.1563 | 0.6902 | -0.5349 | <10-4 | 0.0001 |
| KIF3A | 4.5936 | 1.0905 | 5.0896 | 1.6642 | 0.9025 | -0.1479 | 0.0172 | 0.0512 |
| LCA5 | 2.8887 | 1.1741 | 3.3186 | 1.6868 | 0.8704 | -0.2002 | 0.0449 | 0.1088 |
| LRRK2 | 23.2301 | 9.1363 | 31.9542 | 17.1448 | 0.7270 | -0.4600 | <10-4 | 0.0002 |
| MAK | 1.0254 | 0.9764 | 1.4294 | 1.6659 | 0.7174 | -0.4792 | 0.0454 | 0.1094 |
| MAPKAP1 | 27.7082 | 5.4166 | 33.7661 | 6.5503 | 0.8206 | -0.2853 | <10-4 | <10-4 |
| MYO1D | 13.3407 | 2.8711 | 15.4683 | 3.5970 | 0.8625 | -0.2135 | <10-4 | 0.0001 |
| NEK1 | 3.2624 | 0.9032 | 3.7728 | 1.1597 | 0.8647 | -0.2097 | 0.0010 | 0.0041 |
| NEK6 | 14.3078 | 4.5213 | 18.8999 | 5.2353 | 0.7570 | -0.4016 | <10-4 | <10-4 |
| NEK8 | 3.1001 | 0.9223 | 2.6339 | 0.5985 | 1.1770 | 0.2351 | 0.0001 | 0.0003 |
| NUBP2 | 16.1964 | 5.1301 | 10.9670 | 3.4061 | 1.4768 | 0.5625 | <10-4 | <10-4 |
| OCRL | 6.9044 | 0.8905 | 7.4644 | 0.9635 | 0.9250 | -0.1125 | 0.0001 | 0.0003 |
| ODF2 | 10.0760 | 2.0689 | 11.4634 | 3.3043 | 0.8790 | -0.1861 | 0.0007 | 0.0034 |
| PACS1 | 12.2393 | 2.3050 | 13.3234 | 2.1191 | 0.9186 | -0.1224 | 0.0009 | 0.0040 |
| PCM1 | 13.2404 | 2.6865 | 15.4042 | 4.0629 | 0.8595 | -0.2184 | <10-4 | 0.0002 |
| PDE1C | 2.1341 | 1.0363 | 2.7111 | 1.2622 | 0.7872 | -0.3452 | 0.0008 | 0.0035 |
| PDE6D | 12.5088 | 2.0891 | 11.0827 | 1.6096 | 1.1287 | 0.1746 | <10-4 | <10-4 |
| PDZD7 | 0.8140 | 0.4445 | 0.6373 | 0.2513 | 1.2773 | 0.3531 | 0.0008 | 0.0037 |
| PEX6 | 4.2283 | 1.4260 | 3.6255 | 1.6290 | 1.1663 | 0.2219 | 0.0076 | 0.0250 |
| PIBF1 | 7.0392 | 1.7113 | 7.5940 | 1.7668 | 0.9269 | -0.1094 | 0.0298 | 0.0793 |
| PKD1L1 | 0.1730 | 0.0787 | 0.2373 | 0.1297 | 0.7289 | -0.4562 | 0.0001 | 0.0004 |
| PKD2 | 12.9821 | 3.0222 | 14.0005 | 3.1691 | 0.9273 | -0.1090 | 0.0251 | 0.0698 |
| PMFBP1 | 0.2360 | 0.1300 | 0.2823 | 0.1491 | 0.8359 | -0.2586 | 0.0244 | 0.0691 |
| PRKACA | 16.8390 | 3.1923 | 19.1912 | 2.8849 | 0.8774 | -0.1886 | <10-4 | <10-4 |
| PRKCSH | 66.3566 | 9.3898 | 59.7673 | 9.1210 | 1.1102 | 0.1509 | <10-4 | <10-4 |
| PRPF31 | 19.8552 | 3.0190 | 17.6264 | 2.4417 | 1.1264 | 0.1718 | <10-4 | <10-4 |
| PRPF4 | 9.4405 | 1.4467 | 10.4978 | 1.5846 | 0.8993 | -0.1532 | <10-4 | <10-4 |
| PRPF6 | 28.5441 | 3.9306 | 25.6752 | 3.2287 | 1.1117 | 0.1528 | <10-4 | <10-4 |
| PRPF8 | 27.9962 | 6.5583 | 35.4960 | 8.2570 | 0.7887 | -0.3424 | <10-4 | <10-4 |
| PTPDC1 | 3.0085 | 0.5972 | 3.2583 | 0.6972 | 0.9233 | -0.1151 | 0.0091 | 0.0294 |
| QRICH2 | 1.1325 | 0.5263 | 0.8600 | 0.3951 | 1.3169 | 0.3972 | 0.0001 | 0.0004 |
| RAB10 | 31.9345 | 4.8681 | 34.8867 | 6.0887 | 0.9154 | -0.1276 | 0.0003 | 0.0016 |
| RFX3 | 1.0177 | 0.4709 | 1.5645 | 1.3222 | 0.6505 | -0.6204 | 0.0003 | 0.0013 |
| RP2 | 10.0908 | 2.8520 | 11.7012 | 3.1842 | 0.8624 | -0.2136 | 0.0003 | 0.0017 |
| RPGR | 6.9660 | 2.1657 | 8.2092 | 2.9343 | 0.8486 | -0.2369 | 0.0012 | 0.0049 |
| RPGRIP1L | 0.9249 | 0.5035 | 1.1623 | 0.9924 | 0.7957 | -0.3296 | 0.0418 | 0.1032 |
| RPTOR | 4.3850 | 1.0033 | 5.1842 | 0.9067 | 0.8458 | -0.2416 | <10-4 | <10-4 |
| RSPH3 | 4.4412 | 1.3107 | 6.0566 | 2.1584 | 0.7333 | -0.4475 | <10-4 | <10-4 |
| RTTN | 1.1577 | 0.2560 | 1.2548 | 0.2875 | 0.9226 | -0.1162 | 0.0154 | 0.0466 |
| SCLT1 | 3.1021 | 0.6953 | 3.3256 | 0.6558 | 0.9328 | -0.1003 | 0.0241 | 0.0691 |
| SEC63 | 18.4771 | 3.4244 | 21.0889 | 3.7535 | 0.8761 | -0.1908 | <10-4 | <10-4 |
| SLC9B2 | 1.5048 | 0.7038 | 1.8553 | 0.8658 | 0.8111 | -0.3021 | 0.0027 | 0.0101 |
| SNAP29 | 8.6532 | 1.1136 | 9.5396 | 1.5307 | 0.9071 | -0.1407 | <10-4 | 0.0001 |
| SNRNP200 | 20.7688 | 3.7426 | 25.0815 | 4.7287 | 0.8280 | -0.2722 | <10-4 | <10-4 |
| SPATA7 | 5.2983 | 1.3625 | 4.8794 | 1.3209 | 1.0859 | 0.1188 | 0.0332 | 0.0853 |
| STARD10 | 13.8321 | 4.9149 | 10.5063 | 3.6350 | 1.3166 | 0.3968 | <10-4 | <10-4 |
| SUFU | 3.7490 | 0.7482 | 4.0440 | 0.8050 | 0.9271 | -0.1093 | 0.0100 | 0.0319 |
| TBCCD1 | 4.1939 | 0.6589 | 4.5698 | 0.7419 | 0.9177 | -0.1239 | 0.0003 | 0.0016 |
| TBX6 | 0.6576 | 0.3098 | 0.4909 | 0.2365 | 1.3397 | 0.4219 | <10-4 | 0.0003 |
| TCTEX1D2 | 12.5652 | 8.6678 | 9.7405 | 9.9357 | 1.2900 | 0.3674 | 0.0393 | 0.0977 |
| TCTN3 | 10.7882 | 1.5647 | 10.2699 | 1.3254 | 1.0505 | 0.0710 | 0.0147 | 0.0447 |
| TMEM138 | 9.2210 | 2.3823 | 8.1518 | 2.0555 | 1.1312 | 0.1778 | 0.0011 | 0.0047 |
| TMEM216 | 6.1873 | 1.4740 | 4.9962 | 1.2761 | 1.2384 | 0.3085 | <10-4 | <10-4 |
| TOPORS | 7.2467 | 1.5541 | 7.9993 | 1.6651 | 0.9059 | -0.1425 | 0.0016 | 0.0062 |
| TRAF3IP1 | 7.3772 | 2.7207 | 8.7311 | 4.1031 | 0.8449 | -0.2431 | 0.0087 | 0.0283 |
| TRIM32 | 3.4543 | 0.8388 | 3.8406 | 1.0502 | 0.8994 | -0.1530 | 0.0060 | 0.0205 |
| TSSK4 | 1.1043 | 0.5990 | 1.3598 | 0.4417 | 0.8121 | -0.3003 | 0.0010 | 0.0041 |
| TTBK2 | 0.9974 | 0.4096 | 1.9778 | 1.1475 | 0.5043 | -0.9877 | <10-4 | <10-4 |
| TTC21B | 3.8051 | 0.9435 | 4.7955 | 1.5412 | 0.7935 | -0.3338 | <10-4 | <10-4 |
| TTC23 | 3.8538 | 0.6054 | 4.1301 | 0.6896 | 0.9331 | -0.0999 | 0.0040 | 0.0141 |
| TTC26 | 2.9309 | 1.5308 | 3.8437 | 2.6973 | 0.7625 | -0.3912 | 0.0051 | 0.0179 |
| TTLL11 | 2.0981 | 0.5918 | 2.4035 | 0.6070 | 0.8730 | -0.1960 | 0.0006 | 0.0029 |
| TTLL3 | 12.2778 | 4.3591 | 10.4064 | 3.8612 | 1.1798 | 0.2386 | 0.0020 | 0.0078 |
| TTLL4 | 3.2707 | 0.5651 | 3.4428 | 0.5917 | 0.9500 | -0.0740 | 0.0425 | 0.1044 |
| TTLL5 | 4.2229 | 0.7858 | 5.1191 | 1.1657 | 0.8249 | -0.2777 | <10-4 | <10-4 |
| TTLL7 | 2.0931 | 0.8849 | 2.7975 | 1.3227 | 0.7482 | -0.4185 | <10-4 | 0.0002 |
| TUBA1A | 154.1991 | 42.9954 | 132.4416 | 37.7128 | 1.1643 | 0.2194 | 0.0003 | 0.0014 |
| TUBA1B | 263.9522 | 62.7947 | 244.6846 | 61.1039 | 1.0787 | 0.1094 | 0.0339 | 0.0865 |
| TUBA1C | 67.6897 | 24.0606 | 60.8483 | 17.2622 | 1.1124 | 0.1537 | 0.0251 | 0.0698 |
| TUBA3D | 0.1487 | 0.1488 | 0.2249 | 0.1193 | 0.6613 | -0.5967 | 0.0001 | 0.0007 |
| TUBA4A | 20.4669 | 6.6354 | 16.4088 | 3.9851 | 1.2473 | 0.3188 | <10-4 | <10-4 |
| TUBA8 | 0.9474 | 0.3433 | 0.8286 | 0.3111 | 1.1435 | 0.1934 | 0.0134 | 0.0411 |
| TUBG1 | 8.4885 | 3.6474 | 7.4479 | 1.5039 | 1.1397 | 0.1887 | 0.0102 | 0.0322 |
| UNC119B | 10.8473 | 2.7190 | 12.2837 | 4.2100 | 0.8831 | -0.1794 | 0.0063 | 0.0214 |
| VHL | 10.7873 | 1.4092 | 11.2289 | 1.2828 | 0.9607 | -0.0579 | 0.0253 | 0.0698 |
| WDPCP | 1.6146 | 0.4267 | 1.8700 | 0.5712 | 0.8635 | -0.2118 | 0.0007 | 0.0032 |
| WDR11 | 12.3980 | 2.0620 | 13.9069 | 2.3236 | 0.8915 | -0.1657 | <10-4 | <10-4 |
| WDR19 | 6.2130 | 1.7008 | 7.2464 | 2.8693 | 0.8574 | -0.2220 | 0.0032 | 0.0118 |
| WDR34 | 18.0132 | 7.2040 | 13.5794 | 8.1783 | 1.3265 | 0.4076 | 0.0001 | 0.0006 |
| WDR60 | 8.1844 | 1.8317 | 10.8602 | 3.7295 | 0.7536 | -0.4081 | <10-4 | <10-4 |
| WDR62 | 0.7552 | 0.4903 | 0.6330 | 0.2235 | 1.1929 | 0.2545 | 0.0270 | 0.0724 |
| WTIP | 5.2579 | 1.7228 | 5.8281 | 1.9358 | 0.9022 | -0.1486 | 0.0342 | 0.0868 |
| XPNPEP3 | 2.9899 | 0.7993 | 3.2835 | 0.9656 | 0.9106 | -0.1352 | 0.0245 | 0.0691 |
| ZNF423 | 1.9268 | 1.1816 | 2.4880 | 1.0595 | 0.7744 | -0.3688 | 0.0007 | 0.0033 |

## **Table S4. List of deregulated cilia/ciliopathy-associated genes (n= 327) in COPD whole lung tissues: GSE76925**

|  | **non-COPD** | | **COPD** | |  |  |  |  |
| --- | --- | --- | --- | --- | --- | --- | --- | --- |
| **Genes Ids** | **mean** | **sd** | **mean** | **sd** | **FC** | **log2FC** | **p value** | **FDR p value** |
| ADAM15 | 6.1256 | 0.6462 | 6.4380 | 0.7437 | 0.9515 | -0.0718 | 0.0198 | 0.0801 |
| ADCY5 | 3.2900 | 0.4259 | 3.4962 | 0.5650 | 0.9410 | -0.0877 | 0.0373 | 0.1338 |
| ALG8 | 8.9412 | 0.1919 | 9.1138 | 0.2185 | 0.9811 | -0.0276 | <10-4 | 0.0004 |
| ALMS1 | 5.8173 | 0.6747 | 6.0626 | 0.6686 | 0.9595 | -0.0596 | 0.0490 | 0.1653 |
| ARL13B | 4.4785 | 1.2787 | 3.3903 | 1.0412 | 1.3210 | 0.4016 | <10-4 | <10-4 |
| ARL2BP | 10.8245 | 0.3561 | 10.9823 | 0.3200 | 0.9856 | -0.0209 | 0.0104 | 0.0467 |
| ARSG | 5.7760 | 0.4478 | 6.0850 | 0.4629 | 0.9492 | -0.0752 | 0.0004 | 0.0034 |
| BBS10 | 6.9310 | 1.1050 | 7.3879 | 1.0024 | 0.9382 | -0.0921 | 0.0174 | 0.0743 |
| BBS7 | 4.7551 | 0.8930 | 3.8983 | 0.9475 | 1.2198 | 0.2866 | <10-4 | 0.0001 |
| BBS9 | 7.1713 | 0.4684 | 7.3627 | 0.4747 | 0.9740 | -0.0380 | 0.0298 | 0.1118 |
| C21orf71 | 4.2802 | 0.4637 | 4.5961 | 0.5961 | 0.9313 | -0.1027 | 0.0028 | 0.0169 |
| CALM3 | 12.5993 | 0.2903 | 12.9095 | 0.3744 | 0.9760 | -0.0351 | <10-4 | 0.0001 |
| CATSPER2 | 12.4994 | 0.6669 | 11.5786 | 1.0194 | 1.0795 | 0.1104 | <10-4 | <10-4 |
| CCDC66 | 7.2538 | 0.5277 | 7.4471 | 0.5143 | 0.9740 | -0.0379 | 0.0447 | 0.1556 |
| CCT2 | 9.9067 | 0.3061 | 10.1414 | 0.3715 | 0.9769 | -0.0338 | 0.0005 | 0.0041 |
| CCT3 | 8.9853 | 0.4061 | 8.7521 | 0.4391 | 1.0266 | 0.0379 | 0.0038 | 0.0212 |
| CCT4 | 6.4640 | 0.3041 | 6.8936 | 0.5395 | 0.9377 | -0.0928 | <10-4 | 0.0001 |
| CCT5 | 7.0403 | 0.5675 | 7.2303 | 0.4886 | 0.9737 | -0.0384 | 0.0454 | 0.1561 |
| CCT8 | 11.5508 | 0.2870 | 11.7200 | 0.2729 | 0.9856 | -0.0210 | 0.0011 | 0.0085 |
| CDK10 | 4.1240 | 0.5601 | 4.4458 | 0.8666 | 0.9276 | -0.1084 | 0.0303 | 0.1126 |
| CENPJ | 7.4527 | 0.4790 | 6.9930 | 0.7757 | 1.0657 | 0.0918 | 0.0006 | 0.0051 |
| CEP164 | 6.8857 | 0.8785 | 7.1822 | 0.7756 | 0.9587 | -0.0608 | 0.0473 | 0.1610 |
| CEP78 | 5.9506 | 0.4556 | 6.3835 | 0.5615 | 0.9322 | -0.1013 | <10-4 | 0.0005 |
| CFTR | 7.3570 | 0.8553 | 7.7669 | 0.9485 | 0.9472 | -0.0782 | 0.0175 | 0.0743 |
| DLD | 9.2337 | 0.6060 | 8.8919 | 0.6576 | 1.0384 | 0.0544 | 0.0046 | 0.0240 |
| DNAL1 | 6.4561 | 0.8875 | 5.9432 | 0.9435 | 1.0863 | 0.1194 | 0.0032 | 0.0185 |
| DYX1C1 | 4.2962 | 0.8749 | 3.5765 | 0.9385 | 1.2012 | 0.2645 | <10-4 | 0.0007 |
| DZIP1 | 4.7069 | 0.7649 | 5.0752 | 0.8815 | 0.9274 | -0.1087 | 0.0205 | 0.0806 |
| EFCAB2 | 4.2116 | 1.1375 | 3.2251 | 1.0701 | 1.3059 | 0.3850 | <10-4 | 0.0001 |
| EVC | 2.9083 | 0.3643 | 3.1923 | 0.4297 | 0.9110 | -0.1344 | 0.0003 | 0.0029 |
| EXOC8 | 5.8110 | 1.6450 | 4.2760 | 1.6952 | 1.3590 | 0.4425 | <10-4 | 0.0001 |
| FAM161A | 5.1267 | 1.1072 | 4.4334 | 1.2206 | 1.1564 | 0.2096 | 0.0019 | 0.0127 |
| GALNT11 | 10.2481 | 0.2638 | 10.4634 | 0.2903 | 0.9794 | -0.0300 | 0.0001 | 0.0009 |
| GLI2 | 5.0048 | 1.2146 | 5.7478 | 1.0368 | 0.8707 | -0.1997 | 0.0003 | 0.0030 |
| GLI3 | 5.8302 | 0.7510 | 6.1564 | 0.6059 | 0.9470 | -0.0786 | 0.0070 | 0.0332 |
| HYLS1 | 6.0485 | 0.5878 | 6.5439 | 0.5293 | 0.9243 | -0.1136 | <10-4 | 0.0001 |
| IFT122 | 3.0000 | 0.2438 | 3.1353 | 0.3740 | 0.9568 | -0.0637 | 0.0349 | 0.1283 |
| IFT20 | 8.4464 | 0.9744 | 7.6559 | 1.1226 | 1.1033 | 0.1418 | 0.0001 | 0.0017 |
| IFT52 | 7.0244 | 0.5965 | 7.4466 | 0.6390 | 0.9433 | -0.0842 | 0.0004 | 0.0034 |
| IFT74 | 8.9300 | 0.7901 | 8.2470 | 1.0188 | 1.0828 | 0.1148 | 0.0002 | 0.0023 |
| IQCB1 | 3.8734 | 0.6757 | 3.3457 | 0.6683 | 1.1577 | 0.2113 | <10-4 | 0.0006 |
| KIAA0586 | 6.7167 | 0.2962 | 6.8447 | 0.2971 | 0.9813 | -0.0272 | 0.0207 | 0.0806 |
| KIAA0753 | 6.9960 | 0.4493 | 7.2487 | 0.3824 | 0.9651 | -0.0512 | 0.0008 | 0.0063 |
| KIF27 | 5.3287 | 1.3698 | 4.5617 | 1.1436 | 1.1681 | 0.2242 | 0.0007 | 0.0059 |
| KIF3A | 3.3583 | 0.6103 | 3.0123 | 0.6134 | 1.1148 | 0.1568 | 0.0026 | 0.0158 |
| KIF7 | 4.7010 | 0.7725 | 5.1426 | 0.7998 | 0.9141 | -0.1295 | 0.0030 | 0.0174 |
| LZTFL1 | 9.0570 | 0.4751 | 8.8094 | 0.5491 | 1.0281 | 0.0400 | 0.0125 | 0.0550 |
| MAPK9 | 8.9922 | 0.3380 | 9.1708 | 0.3542 | 0.9805 | -0.0284 | 0.0064 | 0.0306 |
| MXRA8 | 6.2455 | 1.0278 | 6.7528 | 0.9503 | 0.9249 | -0.1127 | 0.0053 | 0.0269 |
| MYO1D | 8.5229 | 0.3274 | 8.9160 | 0.5151 | 0.9559 | -0.0651 | <10-4 | 0.0003 |
| MYO7A | 3.7841 | 0.7508 | 4.4253 | 0.9174 | 0.8551 | -0.2258 | 0.0001 | 0.0016 |
| NEK1 | 8.4268 | 0.5809 | 8.0304 | 0.7266 | 1.0494 | 0.0695 | 0.0022 | 0.0141 |
| NEK6 | 7.6980 | 0.6196 | 8.2120 | 0.5830 | 0.9374 | -0.0933 | <10-4 | 0.0001 |
| NPHP3 | 9.0739 | 0.4944 | 9.4412 | 0.4490 | 0.9611 | -0.0573 | <10-4 | 0.0006 |
| NUBP2 | 5.8793 | 0.6025 | 6.2652 | 0.6628 | 0.9384 | -0.0917 | 0.0015 | 0.0107 |
| OCRL | 9.0493 | 0.2730 | 9.2686 | 0.3441 | 0.9763 | -0.0345 | 0.0004 | 0.0034 |
| ODF2 | 5.4202 | 0.6872 | 5.7517 | 0.6679 | 0.9424 | -0.0856 | 0.0084 | 0.0393 |
| OFD1 | 9.0759 | 0.5226 | 8.5935 | 0.7345 | 1.0561 | 0.0788 | 0.0002 | 0.0023 |
| PACS1 | 7.4078 | 0.5258 | 7.6523 | 0.6788 | 0.9680 | -0.0469 | 0.0407 | 0.1431 |
| PDE4C | 13.8800 | 0.4042 | 13.5458 | 0.4983 | 1.0247 | 0.0352 | 0.0002 | 0.0023 |
| PDE6D | 8.2235 | 0.2725 | 8.3926 | 0.3256 | 0.9799 | -0.0294 | 0.0039 | 0.0212 |
| PKD2 | 8.2197 | 1.3840 | 7.2675 | 1.5067 | 1.1310 | 0.1776 | 0.0006 | 0.0052 |
| PKD2L1 | 5.8404 | 1.4537 | 6.4417 | 1.3951 | 0.9066 | -0.1414 | 0.0222 | 0.0853 |
| PRPF3 | 10.0463 | 0.4126 | 10.2577 | 0.4102 | 0.9794 | -0.0300 | 0.0060 | 0.0295 |
| PRPF31 | 9.9903 | 0.3552 | 10.1285 | 0.2985 | 0.9864 | -0.0198 | 0.0184 | 0.0761 |
| PRPF4 | 9.4075 | 0.2621 | 9.5789 | 0.3142 | 0.9821 | -0.0260 | 0.0024 | 0.0151 |
| PRPF8 | 11.1632 | 0.2443 | 11.3573 | 0.3541 | 0.9829 | -0.0249 | 0.0017 | 0.0112 |
| PTPDC1 | 7.4491 | 0.4054 | 7.6418 | 0.5259 | 0.9748 | -0.0368 | 0.0373 | 0.1338 |
| RAB8A | 10.6868 | 0.2323 | 10.8647 | 0.3334 | 0.9836 | -0.0238 | 0.0022 | 0.0141 |
| RICTOR | 3.6827 | 0.5712 | 3.3471 | 0.5048 | 1.1003 | 0.1379 | 0.0007 | 0.0054 |
| RILPL1 | 9.9591 | 0.3584 | 9.6088 | 0.4821 | 1.0365 | 0.0517 | <10-4 | 0.0007 |
| RPGR | 8.7718 | 0.6124 | 8.4456 | 0.6983 | 1.0386 | 0.0547 | 0.0099 | 0.0449 |
| RTTN | 8.0572 | 0.4461 | 8.2286 | 0.3778 | 0.9792 | -0.0304 | 0.0205 | 0.0806 |
| SDCCAG8 | 6.0612 | 0.6847 | 6.4426 | 0.7310 | 0.9408 | -0.0881 | 0.0046 | 0.0240 |
| SNRNP200 | 7.7906 | 0.4986 | 7.9611 | 0.4183 | 0.9786 | -0.0312 | 0.0376 | 0.1338 |
| SPAG16 | 3.7154 | 0.7725 | 3.0981 | 0.5556 | 1.1992 | 0.2621 | <10-4 | <10-4 |
| SPAG4 | 3.9742 | 1.1342 | 4.6879 | 1.2281 | 0.8478 | -0.2383 | 0.0016 | 0.0109 |
| SPATA7 | 8.7904 | 0.4149 | 8.9942 | 0.4782 | 0.9773 | -0.0331 | 0.0181 | 0.0759 |
| TAPT1 | 8.7880 | 0.5358 | 8.1254 | 0.7783 | 1.0815 | 0.1131 | <10-4 | 0.0001 |
| TCP1 | 8.3036 | 1.3847 | 7.6538 | 1.3335 | 1.0849 | 0.1176 | 0.0098 | 0.0449 |
| TCTN3 | 7.5822 | 0.3174 | 7.7346 | 0.2630 | 0.9803 | -0.0287 | 0.0035 | 0.0196 |
| TMEM107 | 4.9554 | 1.2300 | 4.2911 | 1.2998 | 1.1548 | 0.2077 | 0.0056 | 0.0283 |
| TMEM138 | 8.3359 | 0.4519 | 8.7088 | 0.4199 | 0.9572 | -0.0631 | <10-4 | 0.0001 |
| TMEM216 | 8.9284 | 0.4275 | 9.1930 | 0.3405 | 0.9712 | -0.0421 | 0.0001 | 0.0017 |
| TRIM32 | 7.5069 | 0.4669 | 7.7271 | 0.4806 | 0.9715 | -0.0417 | 0.0134 | 0.0584 |
| TTC30B | 5.7326 | 1.4181 | 4.6357 | 1.4052 | 1.2366 | 0.3064 | <10-4 | 0.0007 |
| TTC8 | 5.3655 | 0.8427 | 4.7821 | 0.9977 | 1.1220 | 0.1661 | 0.0012 | 0.0089 |
| TUBA1A | 11.6631 | 0.5600 | 11.3145 | 0.6857 | 1.0308 | 0.0438 | 0.0045 | 0.0240 |
| TUBA1B | 13.2496 | 0.2103 | 13.0765 | 0.2696 | 1.0132 | 0.0190 | 0.0003 | 0.0033 |
| TUBA3E | 3.2869 | 0.4502 | 2.9029 | 0.3977 | 1.1323 | 0.1792 | <10-4 | 0.0001 |
| TUBA4A | 8.6125 | 1.2918 | 7.7857 | 1.3641 | 1.1062 | 0.1456 | 0.0011 | 0.0083 |
| TUBB | 9.6743 | 0.4056 | 9.8862 | 0.5541 | 0.9786 | -0.0312 | 0.0285 | 0.1084 |
| TUBB2A | 7.6807 | 0.9418 | 6.9798 | 1.0186 | 1.1004 | 0.1380 | 0.0002 | 0.0023 |
| TUBB6 | 9.8572 | 0.6187 | 9.4776 | 0.5233 | 1.0401 | 0.0567 | 0.0003 | 0.0028 |
| VHL | 10.0080 | 0.2655 | 10.1608 | 0.3085 | 0.9850 | -0.0219 | 0.0061 | 0.0298 |
| XPNPEP2 | 4.3086 | 0.7880 | 4.6340 | 0.7245 | 0.9298 | -0.1050 | 0.0186 | 0.0761 |
| ZNF423 | 3.1694 | 0.5117 | 3.5077 | 0.5874 | 0.9035 | -0.1463 | 0.0015 | 0.0107 |

## **Table S5. List of deregulated cilia/ciliopathy-associated genes (n= 396) in COPD whole lung tissues: GSE103174**

|  | **non-COPD** | | **COPD** | |  |  |  |  |
| --- | --- | --- | --- | --- | --- | --- | --- | --- |
| **Genes Ids** | **mean** | **sd** | **mean** | **sd** | **FC** | **log2FC** | **p value** | **FDR p value** |
| AGBL2 | 4.4574 | 0.9784 | 3.8244 | 0.6244 | 1.1655 | 0.2210 | 0.0065 | 0.1995 |
| AK7 | 4.7096 | 0.6826 | 4.2466 | 0.6702 | 1.1090 | 0.1493 | 0.0258 | 0.2755 |
| ARL6 | 5.5728 | 0.4713 | 5.1278 | 0.5855 | 1.0868 | 0.1201 | 0.0098 | 0.2109 |
| ARMC9 | 5.8982 | 0.4572 | 5.5807 | 0.5359 | 1.0569 | 0.0798 | 0.0441 | 0.2843 |
| BBOF1 | 6.5365 | 0.7637 | 6.0049 | 0.6897 | 1.0885 | 0.1224 | 0.0159 | 0.2473 |
| BBS7 | 6.4736 | 0.3441 | 6.1961 | 0.3254 | 1.0448 | 0.0632 | 0.0072 | 0.2024 |
| C21orf59 | 9.2858 | 0.2778 | 9.0951 | 0.3136 | 1.0210 | 0.0299 | 0.0408 | 0.2843 |
| CATSPERD | 4.5821 | 0.7660 | 4.1943 | 0.5165 | 1.0925 | 0.1276 | 0.0357 | 0.2828 |
| CCDC181 | 4.4972 | 0.5920 | 4.1785 | 0.4114 | 1.0763 | 0.1061 | 0.0283 | 0.2755 |
| CCDC65 | 4.8937 | 0.8539 | 4.4592 | 0.6444 | 1.0974 | 0.1342 | 0.0467 | 0.2843 |
| CCP110 | 7.8503 | 0.3207 | 7.5876 | 0.2706 | 1.0346 | 0.0491 | 0.0035 | 0.1956 |
| CEP78 | 6.2699 | 0.4036 | 6.0630 | 0.2581 | 1.0341 | 0.0484 | 0.0292 | 0.2755 |
| CFAP126 | 5.2248 | 1.2994 | 4.4688 | 1.1351 | 1.1692 | 0.2255 | 0.0380 | 0.2843 |
| CFAP157 | 4.7753 | 0.9094 | 4.2152 | 0.6195 | 1.1329 | 0.1800 | 0.0118 | 0.2232 |
| CFAP206 | 5.0746 | 0.8653 | 4.5919 | 0.6511 | 1.1051 | 0.1442 | 0.0296 | 0.2755 |
| CFAP43 | 5.9391 | 1.4435 | 5.0098 | 1.1591 | 1.1855 | 0.2455 | 0.0162 | 0.2473 |
| CFAP52 | 5.7511 | 1.4634 | 4.9218 | 1.2818 | 1.1685 | 0.2247 | 0.0433 | 0.2843 |
| CFAP53 | 5.1254 | 1.3031 | 4.4723 | 0.9559 | 1.1460 | 0.1966 | 0.0465 | 0.2843 |
| CFAP61 | 4.0254 | 0.6129 | 3.6655 | 0.4338 | 1.0982 | 0.1351 | 0.0183 | 0.2595 |
| CFAP69 | 6.6405 | 0.5361 | 6.2501 | 0.6194 | 1.0625 | 0.0874 | 0.0332 | 0.2755 |
| CFAP70 | 6.3427 | 1.2034 | 5.6664 | 0.8638 | 1.1194 | 0.1627 | 0.0246 | 0.2755 |
| CFAP73 | 5.1866 | 0.7717 | 4.6281 | 0.4785 | 1.1207 | 0.1644 | 0.0023 | 0.1718 |
| CFAP77 | 4.4784 | 0.7668 | 4.0473 | 0.3963 | 1.1065 | 0.1460 | 0.0093 | 0.2109 |
| CSPP1 | 7.5434 | 0.4450 | 7.2135 | 0.4210 | 1.0457 | 0.0645 | 0.0130 | 0.2313 |
| DNAH10 | 5.4110 | 0.8057 | 4.9753 | 0.6779 | 1.0876 | 0.1211 | 0.0477 | 0.2843 |
| DNAH11 | 6.3704 | 0.4795 | 5.9917 | 0.4230 | 1.0632 | 0.0884 | 0.0059 | 0.1995 |
| DNAH12 | 5.5001 | 1.5415 | 4.5768 | 1.1440 | 1.2018 | 0.2651 | 0.0190 | 0.2595 |
| DNAH2 | 6.2567 | 1.6988 | 5.3245 | 1.2760 | 1.1751 | 0.2328 | 0.0321 | 0.2755 |
| DNAH3 | 3.5856 | 0.4852 | 3.2754 | 0.2258 | 1.0947 | 0.1305 | 0.0024 | 0.1718 |
| DNAH5 | 6.0868 | 1.1068 | 5.4135 | 1.0117 | 1.1244 | 0.1691 | 0.0353 | 0.2828 |
| DNAH6 | 5.0935 | 0.7316 | 4.5954 | 0.3124 | 1.1084 | 0.1485 | 0.0010 | 0.1718 |
| DNAH7 | 5.1081 | 1.1822 | 4.4908 | 0.9287 | 1.1375 | 0.1858 | 0.0462 | 0.2843 |
| DNAI2 | 4.7927 | 1.1894 | 4.0634 | 0.6760 | 1.1795 | 0.2382 | 0.0065 | 0.1995 |
| DRC1 | 4.5690 | 1.0468 | 4.0019 | 0.6719 | 1.1417 | 0.1912 | 0.0217 | 0.2755 |
| DRC3 | 5.7325 | 1.0988 | 4.8325 | 0.8286 | 1.1862 | 0.2464 | 0.0019 | 0.1718 |
| DRC7 | 4.5042 | 0.6229 | 4.0279 | 0.4431 | 1.1182 | 0.1612 | 0.0026 | 0.1718 |
| DZIP1L | 5.7296 | 0.5062 | 5.3743 | 0.4493 | 1.0661 | 0.0924 | 0.0140 | 0.2313 |
| FOXJ1 | 4.7623 | 1.1548 | 4.0412 | 0.7607 | 1.1784 | 0.2369 | 0.0096 | 0.2109 |
| IFT172 | 6.5420 | 0.5961 | 6.2002 | 0.4885 | 1.0551 | 0.0774 | 0.0334 | 0.2755 |
| IFT22 | 7.7512 | 0.3275 | 7.4178 | 0.5915 | 1.0449 | 0.0634 | 0.0397 | 0.2843 |
| IFT74 | 7.6366 | 0.3541 | 7.3721 | 0.4199 | 1.0359 | 0.0509 | 0.0323 | 0.2755 |
| IFT88 | 6.6647 | 0.4089 | 6.4137 | 0.3721 | 1.0391 | 0.0554 | 0.0332 | 0.2755 |
| IQCA1 | 6.8799 | 0.6895 | 6.2978 | 0.8696 | 1.0924 | 0.1275 | 0.0216 | 0.2755 |
| IQCG | 6.2851 | 0.7292 | 5.8303 | 0.7287 | 1.0780 | 0.1084 | 0.0421 | 0.2843 |
| IQUB | 3.8168 | 0.4942 | 3.5690 | 0.3148 | 1.0694 | 0.0969 | 0.0324 | 0.2755 |
| KIF24 | 3.9786 | 0.4570 | 3.6776 | 0.2347 | 1.0818 | 0.1135 | 0.0025 | 0.1718 |
| LZTFL1 | 7.3276 | 0.2701 | 6.9944 | 0.5754 | 1.0476 | 0.0671 | 0.0320 | 0.2755 |
| MAATS1 | 4.9239 | 0.7963 | 4.4628 | 0.6100 | 1.1033 | 0.1419 | 0.0256 | 0.2755 |
| MAPK9 | 7.2608 | 0.3106 | 7.1003 | 0.2416 | 1.0226 | 0.0323 | 0.0472 | 0.2843 |
| NEK6 | 7.0815 | 0.4293 | 7.5055 | 0.5143 | 0.9435 | -0.0839 | 0.0057 | 0.1995 |
| OFD1 | 8.7771 | 0.3678 | 8.5835 | 0.2753 | 1.0226 | 0.0322 | 0.0390 | 0.2843 |
| PIFO | 7.3731 | 1.2142 | 6.6546 | 1.1658 | 1.1080 | 0.1479 | 0.0471 | 0.2843 |
| PRPF4 | 7.6627 | 0.2658 | 7.8580 | 0.2349 | 0.9751 | -0.0363 | 0.0101 | 0.2109 |
| RP1 | 5.7950 | 1.4635 | 4.8619 | 0.8343 | 1.1919 | 0.2533 | 0.0049 | 0.1995 |
| SNTN | 6.3182 | 1.9539 | 5.1339 | 1.9707 | 1.2307 | 0.2995 | 0.0493 | 0.2843 |
| SPAG17 | 4.8374 | 1.0497 | 4.0790 | 0.8094 | 1.1859 | 0.2460 | 0.0062 | 0.1995 |
| TCTN1 | 8.6041 | 0.3773 | 8.3638 | 0.3076 | 1.0287 | 0.0409 | 0.0184 | 0.2595 |
| TEKT2 | 4.9609 | 1.1866 | 4.2558 | 0.9629 | 1.1657 | 0.2212 | 0.0268 | 0.2755 |
| TMEM107 | 7.0162 | 0.2881 | 6.8454 | 0.2378 | 1.0250 | 0.0356 | 0.0287 | 0.2755 |
| TMEM231 | 6.5107 | 0.8040 | 5.8830 | 0.7386 | 1.1067 | 0.1463 | 0.0079 | 0.2082 |
| TRAF3IP1 | 5.3510 | 0.4085 | 5.1315 | 0.3367 | 1.0428 | 0.0604 | 0.0464 | 0.2843 |
| TTLL6 | 3.3394 | 0.2371 | 3.1758 | 0.1941 | 1.0515 | 0.0725 | 0.0112 | 0.2214 |
| TULP3 | 7.6016 | 0.3201 | 7.3912 | 0.3055 | 1.0285 | 0.0405 | 0.0275 | 0.2755 |
| WDPCP | 5.4044 | 0.4610 | 5.0709 | 0.4273 | 1.0658 | 0.0919 | 0.0139 | 0.2313 |
| WDR62 | 4.3117 | 0.3118 | 4.1272 | 0.2932 | 1.0447 | 0.0631 | 0.0441 | 0.2843 |
| ZMYND10 | 5.2379 | 1.3501 | 4.4363 | 1.1167 | 1.1807 | 0.2396 | 0.0287 | 0.2755 |

## **Table S6. List of deregulated cilia/ciliopathy-associated genes (n= 398) in COPD SAEC: GSE11784**

|  | **non-COPD** | | **COPD** | |  |  |  |  |
| --- | --- | --- | --- | --- | --- | --- | --- | --- |
| **Genes Ids** | **mean** | **sd** | **mean** | **sd** | **FC** | **log2FC** | **p value** | **FDR p value** |
| ABHD2 | 1041.7978 | 529.0864 | 1572.9700 | 478.1904 | 0.6623 | -0.5944 | <10-4 | 0.0012 |
| AGBL5 | 651.9084 | 233.3230 | 824.2607 | 185.9684 | 0.7909 | -0.3384 | 0.0012 | 0.0220 |
| AKAP14 | 9786.8228 | 3249.7870 | 11930.9836 | 2297.7255 | 0.8203 | -0.2858 | 0.0034 | 0.0401 |
| AKAP4 | 32.4454 | 26.8230 | 50.7833 | 33.4475 | 0.6389 | -0.6463 | 0.0047 | 0.0469 |
| ANKMY2 | 3929.0699 | 1340.2052 | 4991.8845 | 1039.9111 | 0.7871 | -0.3454 | 0.0005 | 0.0129 |
| ANXA1 | 22087.4006 | 6464.0795 | 26229.9409 | 4593.7059 | 0.8421 | -0.2480 | 0.0045 | 0.0455 |
| CABYR | 407.2802 | 457.0470 | 1074.9003 | 819.2747 | 0.3789 | -1.4001 | <10-4 | <10-4 |
| CATSPER3 | 195.2467 | 76.5907 | 265.7082 | 82.2405 | 0.7348 | -0.4445 | 0.0001 | 0.0041 |
| CCDC151 | 3365.2190 | 1274.9667 | 4283.0714 | 1377.3190 | 0.7857 | -0.3479 | 0.0023 | 0.0309 |
| CCDC28B | 46.1573 | 31.0820 | 69.5856 | 36.9297 | 0.6633 | -0.5922 | 0.0017 | 0.0263 |
| CCT4 | 6234.4003 | 1737.1415 | 7583.8291 | 1477.1681 | 0.8221 | -0.2827 | 0.0007 | 0.0163 |
| CCT5 | 3346.1379 | 1262.7640 | 4507.9027 | 1057.9478 | 0.7423 | -0.4300 | 0.0001 | 0.0028 |
| DCTN1 | 821.7683 | 311.2504 | 1058.8436 | 352.2979 | 0.7761 | -0.3657 | 0.0014 | 0.0224 |
| DNAI2 | 7578.4588 | 2743.8580 | 10480.2745 | 2449.5799 | 0.7231 | -0.4677 | <10-4 | 0.0007 |
| EHD1 | 281.6501 | 105.3724 | 357.3736 | 144.9142 | 0.7881 | -0.3435 | 0.0036 | 0.0403 |
| IFT20 | 3503.9721 | 1043.0651 | 4175.9086 | 731.6164 | 0.8391 | -0.2531 | 0.0042 | 0.0443 |
| IQCD | 1318.3822 | 404.2988 | 1623.3465 | 371.4205 | 0.8121 | -0.3002 | 0.0011 | 0.0216 |
| KATNB1 | 2430.1975 | 930.2024 | 3286.3664 | 785.0393 | 0.7395 | -0.4354 | 0.0001 | 0.0028 |
| KIAA0586 | 366.6183 | 132.7450 | 482.6555 | 147.8165 | 0.7596 | -0.3967 | 0.0003 | 0.0073 |
| LRGUK | 1041.8511 | 328.6757 | 1279.0958 | 329.8017 | 0.8145 | -0.2960 | 0.0020 | 0.0285 |
| LYZL4 | 96.0547 | 46.8171 | 139.0566 | 62.8173 | 0.6908 | -0.5337 | 0.0002 | 0.0065 |
| MAPKAP1 | 568.7635 | 209.8021 | 710.7184 | 167.9572 | 0.8003 | -0.3214 | 0.0030 | 0.0371 |
| NEK6 | 674.6443 | 324.4911 | 1092.8196 | 355.0948 | 0.6173 | -0.6959 | <10-4 | <10-4 |
| PIH1D3 | 4775.8595 | 1798.1921 | 5979.8477 | 1715.8057 | 0.7987 | -0.3243 | 0.0039 | 0.0420 |
| PROM2 | 1139.0961 | 503.7590 | 1663.5250 | 572.9007 | 0.6847 | -0.5464 | <10-4 | 0.0012 |
| RAB10 | 5649.9529 | 1949.1090 | 7052.2782 | 1135.9289 | 0.8012 | -0.3199 | 0.0013 | 0.0222 |
| RILPL2 | 2314.6676 | 702.8938 | 2998.3518 | 874.8971 | 0.7720 | -0.3734 | 0.0001 | 0.0028 |
| ROPN1L | 10253.8957 | 3758.7509 | 13029.7014 | 2748.3753 | 0.7870 | -0.3456 | 0.0011 | 0.0216 |
| RSPH9 | 6175.0442 | 2098.7969 | 7542.3023 | 1313.0345 | 0.8187 | -0.2886 | 0.0036 | 0.0403 |
| SCNN1A | 7244.0549 | 2570.2537 | 9325.5191 | 1881.0551 | 0.7768 | -0.3644 | 0.0004 | 0.0099 |
| SNAP29 | 707.7757 | 263.7699 | 938.1220 | 232.8257 | 0.7545 | -0.4065 | 0.0002 | 0.0056 |
| TCTEX1D2 | 5806.8236 | 2027.8684 | 7228.0864 | 1583.5806 | 0.8034 | -0.3159 | 0.0021 | 0.0285 |
| TMEM216 | 1460.8350 | 427.9327 | 1792.0005 | 312.7058 | 0.8152 | -0.2948 | 0.0007 | 0.0154 |
| TUBB | 3292.8346 | 1346.0243 | 4223.8318 | 1375.0070 | 0.7796 | -0.3592 | 0.0032 | 0.0380 |
| TUBB2A | 5123.9018 | 2045.2141 | 7596.5082 | 2062.9588 | 0.6745 | -0.5681 | <10-4 | 0.0001 |
| TUBB4B | 27107.4248 | 8680.5501 | 33656.4909 | 6770.3587 | 0.8054 | -0.3122 | 0.0009 | 0.0197 |
| TUBB6 | 251.5315 | 209.9793 | 408.9273 | 262.0143 | 0.6151 | -0.7011 | 0.0020 | 0.0285 |
| TUBG1 | 609.2672 | 230.4694 | 832.9294 | 276.9465 | 0.7315 | -0.4511 | 0.0001 | 0.0028 |
| WDR34 | 3024.2750 | 1264.8826 | 3984.2523 | 1400.1587 | 0.7591 | -0.3977 | 0.0014 | 0.0224 |
| ZMYND10 | 3929.1584 | 1151.0569 | 4756.7750 | 1359.2583 | 0.8260 | -0.2758 | 0.0027 | 0.0349 |

## **Table S7. List of deregulated cilia/ciliopathy-associated genes (n= 154) in COPD SAEC: GSE37147**

|  | **non-COPD** | | **COPD** | |  |  |  |  |
| --- | --- | --- | --- | --- | --- | --- | --- | --- |
| **Genes Ids** | **mean** | **sd** | **mean** | **sd** | **FC** | **log2FC** | **p value** | **FDR p value** |
| ADAM15 | 6.3774 | 0.2553 | 6.5392 | 0.2438 | 0.9753 | -0.0361 | <10-4 | 0.0002 |
| ADCY6 | 6.9175 | 0.1896 | 7.0213 | 0.1439 | 0.9852 | -0.0215 | <10-4 | 0.0004 |
| AK7 | 9.0811 | 0.2486 | 8.9815 | 0.2291 | 1.0111 | 0.0159 | 0.0024 | 0.0157 |
| ANKMY2 | 7.5872 | 0.2746 | 7.5107 | 0.2517 | 1.0102 | 0.0146 | 0.0339 | 0.1244 |
| ANKS6 | 6.7131 | 0.1805 | 6.7972 | 0.1908 | 0.9876 | -0.0180 | 0.0008 | 0.0090 |
| ARMC4 | 9.6099 | 0.2253 | 9.4937 | 0.2375 | 1.0122 | 0.0175 | 0.0002 | 0.0028 |
| BBS10 | 6.5981 | 0.3437 | 6.4483 | 0.3276 | 1.0232 | 0.0332 | 0.0011 | 0.0102 |
| BBS12 | 6.7525 | 0.2229 | 6.6935 | 0.2065 | 1.0088 | 0.0127 | 0.0446 | 0.1437 |
| BBS2 | 8.3984 | 0.1492 | 8.3120 | 0.1630 | 1.0104 | 0.0149 | <10-4 | 0.0009 |
| BBS4 | 8.3994 | 0.2452 | 8.2495 | 0.2246 | 1.0182 | 0.0260 | <10-4 | 0.0002 |
| BBS7 | 6.4936 | 0.2407 | 6.4293 | 0.2218 | 1.0100 | 0.0144 | 0.0423 | 0.1437 |
| C5orf30 | 6.1228 | 0.4972 | 5.9670 | 0.4968 | 1.0261 | 0.0372 | 0.0207 | 0.0838 |
| C8orf37 | 6.6929 | 0.3202 | 6.5523 | 0.2911 | 1.0214 | 0.0306 | 0.0009 | 0.0090 |
| CCDC39 | 8.7130 | 0.3307 | 8.6279 | 0.2895 | 1.0099 | 0.0142 | 0.0468 | 0.1471 |
| CCNO | 6.4440 | 0.2442 | 6.5237 | 0.2038 | 0.9878 | -0.0177 | 0.0108 | 0.0493 |
| CIB2 | 3.8228 | 0.1730 | 3.8804 | 0.1962 | 0.9852 | -0.0216 | 0.0195 | 0.0810 |
| CUL3 | 8.4691 | 0.1647 | 8.4133 | 0.1560 | 1.0066 | 0.0095 | 0.0110 | 0.0493 |
| DCDC2 | 7.5276 | 0.2927 | 7.3652 | 0.3208 | 1.0220 | 0.0315 | 0.0001 | 0.0016 |
| EFCAB2 | 8.8841 | 0.3929 | 8.7128 | 0.3792 | 1.0197 | 0.0281 | 0.0012 | 0.0102 |
| EFCAB7 | 6.7764 | 0.2985 | 6.6718 | 0.2501 | 1.0157 | 0.0224 | 0.0063 | 0.0346 |
| EXOC4 | 8.8715 | 0.1215 | 8.8371 | 0.1111 | 1.0039 | 0.0056 | 0.0311 | 0.1197 |
| GPR157 | 5.8786 | 0.1944 | 5.9567 | 0.1840 | 0.9869 | -0.0190 | 0.0026 | 0.0160 |
| GPR161 | 5.1211 | 0.1834 | 5.2250 | 0.1870 | 0.9801 | -0.0290 | <10-4 | 0.0009 |
| KIF14 | 3.2814 | 0.1988 | 3.3673 | 0.2969 | 0.9745 | -0.0373 | 0.0081 | 0.0411 |
| KIF3A | 8.3077 | 0.2259 | 8.2465 | 0.1974 | 1.0074 | 0.0107 | 0.0363 | 0.1301 |
| LRRC56 | 6.5266 | 0.2671 | 6.6337 | 0.2445 | 0.9839 | -0.0235 | 0.0024 | 0.0157 |
| LRRC6 | 9.0783 | 0.2427 | 9.0145 | 0.2170 | 1.0071 | 0.0102 | 0.0435 | 0.1437 |
| MAPK9 | 7.4659 | 0.1830 | 7.3872 | 0.1866 | 1.0107 | 0.0153 | 0.0017 | 0.0132 |
| MAPKBP1 | 6.2087 | 0.1796 | 6.2735 | 0.1702 | 0.9897 | -0.0150 | 0.0068 | 0.0360 |
| PACS1 | 7.0861 | 0.2855 | 7.1676 | 0.2788 | 0.9886 | -0.0165 | 0.0335 | 0.1244 |
| PROM1 | 10.5104 | 0.2337 | 10.5762 | 0.2707 | 0.9938 | -0.0090 | 0.0499 | 0.1508 |
| PROM2 | 6.2719 | 0.2904 | 6.4585 | 0.3272 | 0.9711 | -0.0423 | <10-4 | 0.0003 |
| QRICH2 | 5.1674 | 0.1858 | 5.2483 | 0.2029 | 0.9846 | -0.0224 | 0.0020 | 0.0145 |
| SDCCAG8 | 7.2838 | 0.1768 | 7.2240 | 0.1685 | 1.0083 | 0.0119 | 0.0112 | 0.0493 |
| SPAG6 | 10.5475 | 0.1898 | 10.4142 | 0.2140 | 1.0128 | 0.0183 | <10-4 | 0.0002 |
| TAPT1 | 7.3819 | 0.2240 | 7.2443 | 0.2434 | 1.0190 | 0.0271 | <10-4 | 0.0004 |
| TCTN1 | 8.8863 | 0.1561 | 8.8415 | 0.1719 | 1.0051 | 0.0073 | 0.0412 | 0.1437 |
| TCTN2 | 9.4521 | 0.2356 | 9.3871 | 0.2459 | 1.0069 | 0.0100 | 0.0448 | 0.1437 |
| TCTN3 | 7.6432 | 0.1982 | 7.5894 | 0.2059 | 1.0071 | 0.0102 | 0.0481 | 0.1482 |
| TEKT1 | 9.6897 | 0.2050 | 9.5951 | 0.2260 | 1.0099 | 0.0142 | 0.0011 | 0.0102 |
| TEKT3 | 6.9907 | 0.3234 | 6.8512 | 0.3542 | 1.0204 | 0.0291 | 0.0022 | 0.0155 |
| TMEM107 | 7.6867 | 0.2455 | 7.5913 | 0.2564 | 1.0126 | 0.0180 | 0.0049 | 0.0280 |
| TMEM138 | 7.7070 | 0.1651 | 7.6569 | 0.1593 | 1.0065 | 0.0094 | 0.0231 | 0.0913 |
| TMEM67 | 10.0045 | 0.2039 | 9.9271 | 0.2355 | 1.0078 | 0.0112 | 0.0083 | 0.0411 |
| TTC23 | 5.9963 | 0.1784 | 5.9330 | 0.1809 | 1.0107 | 0.0153 | 0.0092 | 0.0444 |
| TTC25 | 8.8848 | 0.2020 | 8.8091 | 0.1866 | 1.0086 | 0.0123 | 0.0046 | 0.0271 |
| TTC26 | 9.0393 | 0.2272 | 8.9250 | 0.2293 | 1.0128 | 0.0184 | 0.0002 | 0.0029 |
| TTC8 | 7.4610 | 0.1977 | 7.3620 | 0.1846 | 1.0134 | 0.0193 | 0.0002 | 0.0025 |
| WDR19 | 9.1857 | 0.2122 | 9.0715 | 0.2277 | 1.0126 | 0.0180 | 0.0001 | 0.0020 |
| WDR34 | 7.7605 | 0.2077 | 7.8246 | 0.1688 | 0.9918 | -0.0119 | 0.0149 | 0.0639 |
| WDR35 | 8.6280 | 0.2244 | 8.5306 | 0.2254 | 1.0114 | 0.0164 | 0.0015 | 0.0119 |

## **Table S8. List of deregulated cilia/ciliopathy-associated genes (n= 419) in COPD SAEC: GSE56341**

|  | **non-COPD** | | **COPD** | |  |  |  |  |
| --- | --- | --- | --- | --- | --- | --- | --- | --- |
| **Genes Ids** | **mean** | **sd** | **mean** | **sd** | **FC** | **log2FC** | **p value** | **FDR p value** |
| ADAM15 | 6.8065 | 0.2187 | 7.1669 | 0.2465 | 0.9497 | -0.0744 | 0.0020 | 0.8356 |
| ADCY6 | 6.9458 | 0.1313 | 7.0578 | 0.0954 | 0.9841 | -0.0231 | 0.0480 | 0.9355 |
| ARL13B | 8.6720 | 0.1267 | 8.4980 | 0.2112 | 1.0205 | 0.0292 | 0.0245 | 0.9355 |
| ARMC4 | 9.7386 | 0.1767 | 9.5833 | 0.1161 | 1.0162 | 0.0232 | 0.0384 | 0.9355 |
| ARSG | 5.7978 | 0.2145 | 5.6230 | 0.1098 | 1.0311 | 0.0442 | 0.0452 | 0.9355 |
| BBS4 | 8.8756 | 0.1344 | 8.6764 | 0.2392 | 1.0230 | 0.0327 | 0.0203 | 0.9355 |
| CALM3 | 8.5455 | 0.1318 | 8.7602 | 0.2653 | 0.9755 | -0.0358 | 0.0189 | 0.9355 |
| CCDC114 | 8.7412 | 0.2678 | 8.9745 | 0.1950 | 0.9740 | -0.0380 | 0.0439 | 0.9355 |
| CCDC181 | 7.3634 | 0.2319 | 7.0597 | 0.3231 | 1.0430 | 0.0608 | 0.0186 | 0.9355 |
| CDK10 | 6.6164 | 0.2101 | 6.8380 | 0.1115 | 0.9676 | -0.0475 | 0.0123 | 0.9355 |
| DCDC2 | 7.3341 | 0.1848 | 7.1074 | 0.2686 | 1.0319 | 0.0453 | 0.0293 | 0.9355 |
| DRC1 | 8.5679 | 0.1307 | 8.7292 | 0.1852 | 0.9815 | -0.0269 | 0.0266 | 0.9355 |
| EFCAB2 | 8.7671 | 0.2705 | 8.4848 | 0.3200 | 1.0333 | 0.0472 | 0.0393 | 0.9355 |
| EHD3 | 4.8342 | 0.2536 | 5.0562 | 0.1601 | 0.9561 | -0.0648 | 0.0380 | 0.9355 |
| ICK | 7.6826 | 0.1400 | 7.8091 | 0.0785 | 0.9838 | -0.0236 | 0.0298 | 0.9355 |
| PKD2 | 8.4001 | 0.1606 | 8.1088 | 0.3837 | 1.0359 | 0.0509 | 0.0206 | 0.9355 |
| PROM2 | 6.0213 | 0.2121 | 6.2565 | 0.1766 | 0.9624 | -0.0553 | 0.0154 | 0.9355 |
| STK36 | 8.1003 | 0.2614 | 8.4035 | 0.2595 | 0.9639 | -0.0530 | 0.0163 | 0.9355 |
| TTC23 | 6.2547 | 0.1777 | 6.0720 | 0.1597 | 1.0301 | 0.0428 | 0.0261 | 0.9355 |
| UBXN10 | 10.6901 | 0.1205 | 10.5832 | 0.1037 | 1.0101 | 0.0145 | 0.0488 | 0.9355 |

# **2. Supporting information Figures**

## **Figure S1. Localization and single-cell transcriptomic signatures of the main cilia hits in COPD whole lung tissues.**

Representative micrographs showing the bronchial epithelia on FFPE lung tissues from the Human Protein Atlas and cellular landscape along the airways in human lung analysed by single-cell RNA sequencing to display specific cluster assignment of epithelial and non-epithelial cells (the identification of each specific gene-expressing cells by t-distributed Stochastic Neighbour Embedding (tSNE) is shown) for Bbs7, Bbs9, Calm3, Cep78, Dzip1, Gli2, Nek6, Nubp2, Pde6d, Prpf4, Prpf8, Ptpdc1, Tmem138, and Wdpcp.

**
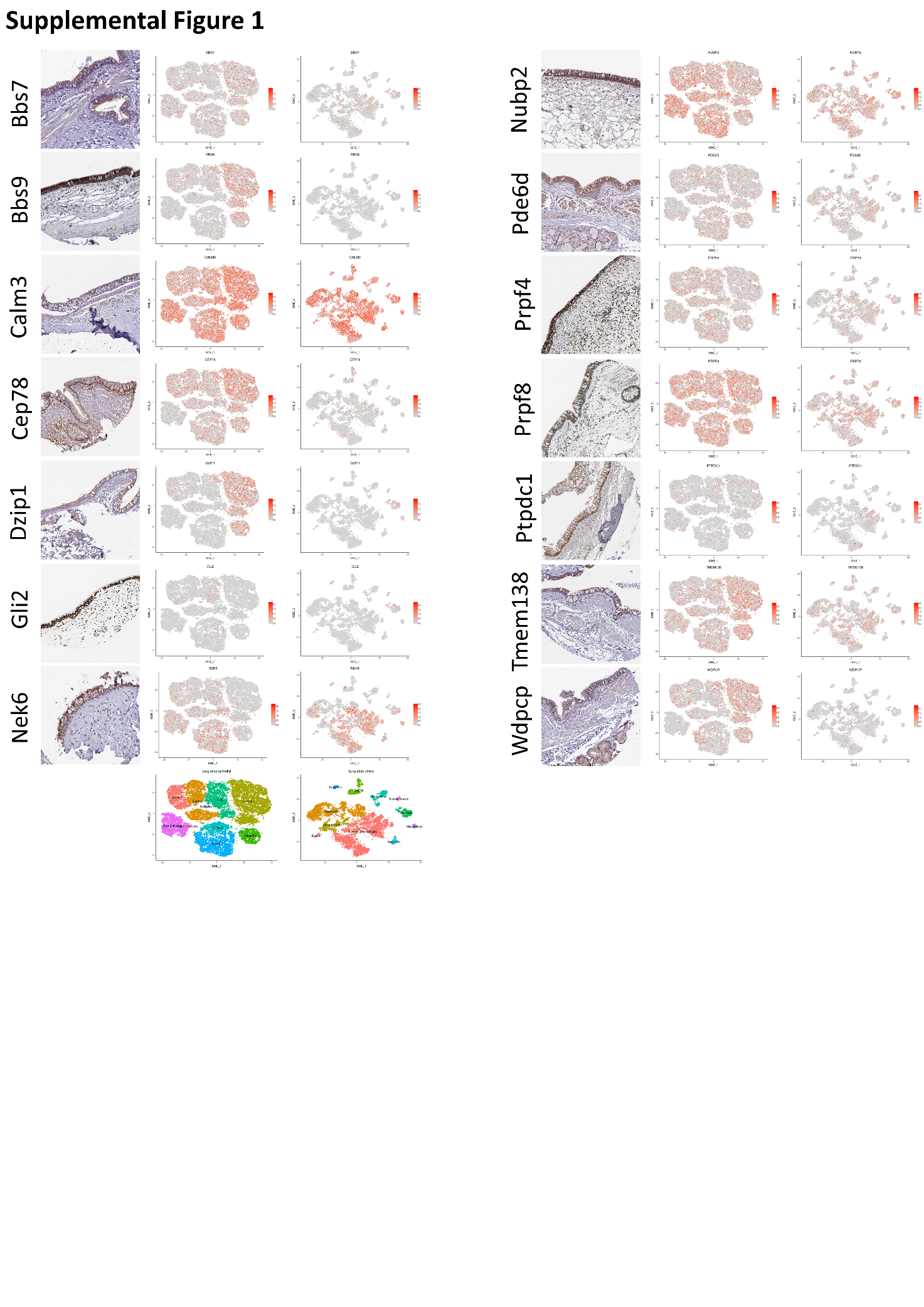
**

## **Figure S2. Localization and single-cell transcriptomic signatures of the main cilia hits in COPD SAEC.**

Representative micrographs showing the bronchial epithelia on FFPE lung tissues from the Human Protein Atlas and cellular landscape along the airways in human lung analysed by single-cell RNA sequencing to display specific cluster assignment of epithelial and non-epithelial cells (the identification of each specific gene-expressing cells by t-distributed Stochastic Neighbour Embedding (tSNE) is shown) for Adam15, Adcy6, Ankmy2, Armc4, Bbs4, Dcdc2, Efcab2, Prom2, Ttc23, Wdr34.


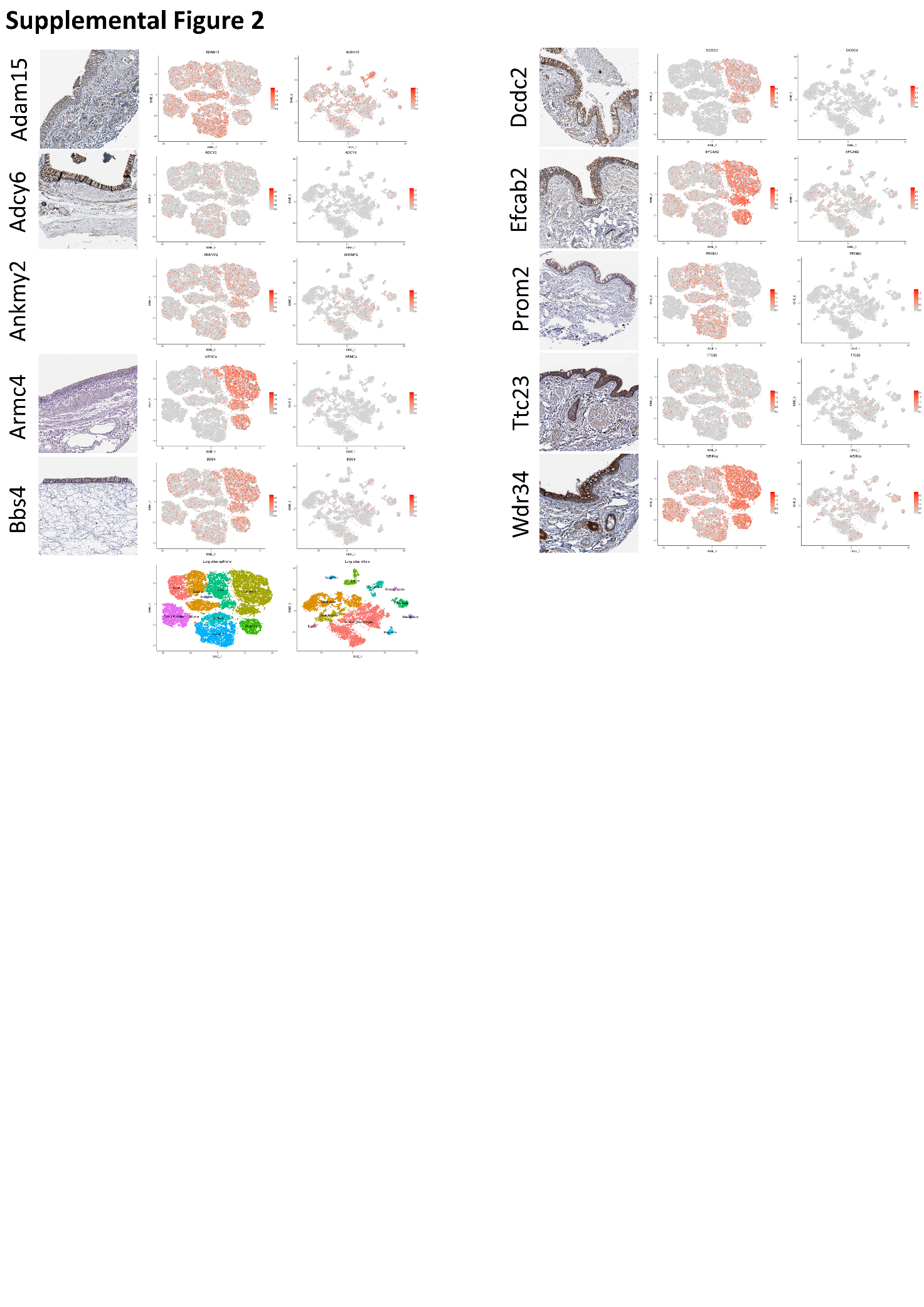


## **Figure S3. Common cilia and ciliopathy-associated deregulated genes signature in whole lung and SAEC.**

**A**, Venn diagram comparing the number of differentially expressed genes shared between whole lung and SAEC datasets. **B**, Table summarizing the log2 of the fold change (when statistically significant) for the 23 commonly dysregulated cilia-associated genes across the 7 datasets. Red highlighting, downregulation in COPD patients; blue highlighting, upregulation in COPD patients. **C**, Venn diagram comparing the number of differentially expressed genes associated to ciliopathies shared between whole lung and SAEC datasets. The selection includes 100 differentially expressed genes in at least 2 datasets for the whole lung and 100 differentially expressed genes in at least 1 dataset for SAEC.


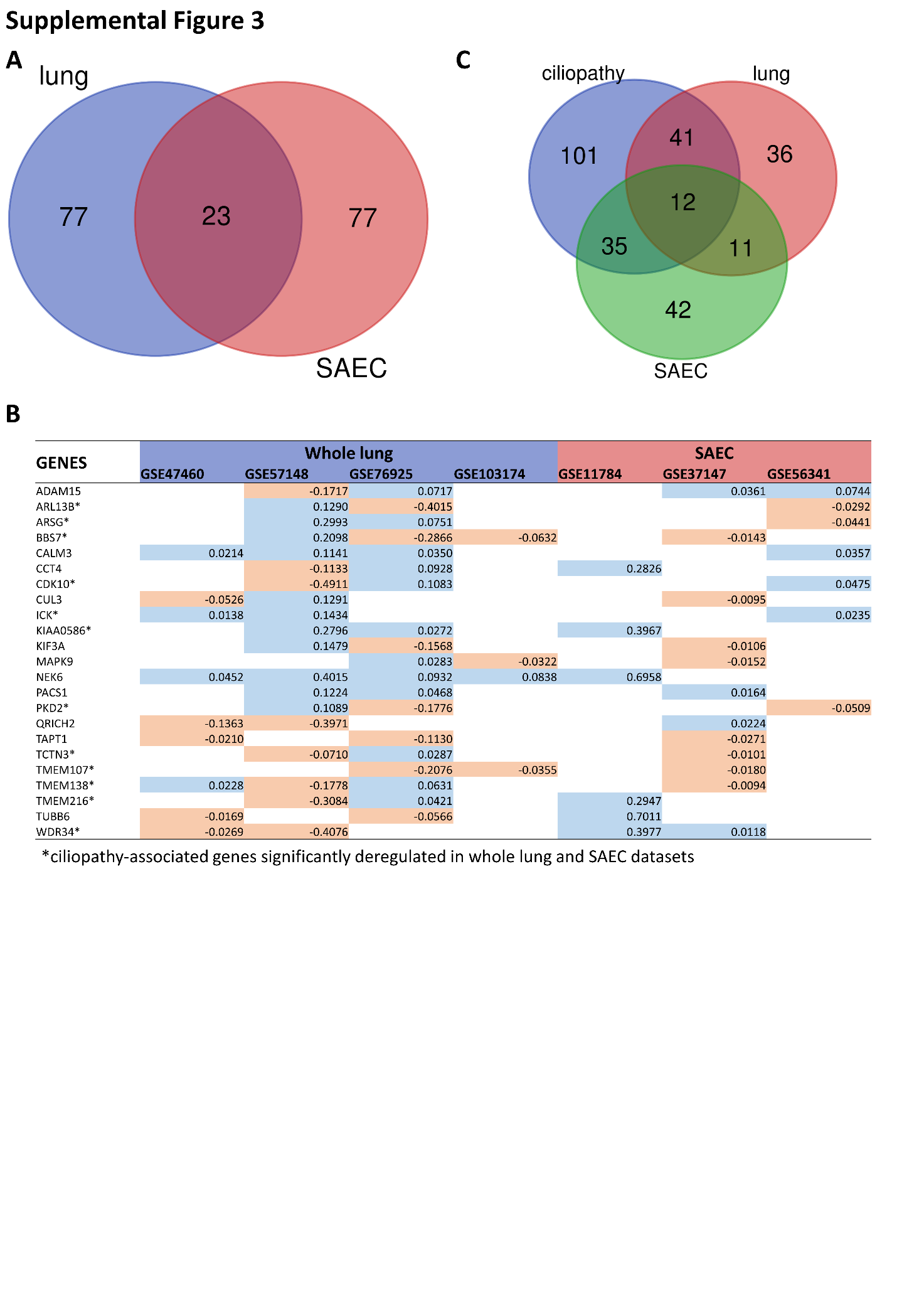

Supplement: Supplementary file 1 — Additional file 1. Additional figures and tables. [file 12931_2021_1665_MOESM1_ESM.docx]
